# *Dcf1* induces glioblastoma cells apoptosis by blocking autophagy

**DOI:** 10.1002/cam4.4440

**Published:** 2021-11-19

**Authors:** Guanghong Luo, Ruili Feng, Wengang Li, Yanlu Chen, Yangyang Sun, Junfeng Ma, Yanhong Duo, Tieqiao Wen

**Affiliations:** ^1^ Laboratory of Molecular Neural Biology School of Life Sciences Shanghai University Shanghai China; ^2^ Department of Radiation Oncology The Second Clinical Medical College Jinan University (Shenzhen People's Hospital) Shenzhen China; ^3^ Integrated Chinese and Western Medicine Postdoctoral Research Station Jinan University Guangzhou China; ^4^ Department of Neurosurgery Shanghai Fifth People's Hospital Fudan University Shanghai China; ^5^ Department of Microbiology, Tumor and Cell Biology (MTC) Karolinska Institutet Stockholm Sweden

**Keywords:** apoptosis, *Dcf1*, glioblastoma, lysosome, mitochondria, mitophagy

## Abstract

**Background:**

*Dcf1* has been demonstrated to play vital roles in many CNS diseases, it also has a destructive role on cell mitochondria in glioma cells and promotes the autophagy. Hitherto, it is unclear whether the viability of glioblastoma cells is affected by *Dcf1*, in particular *Dcf1* possesses broad localization on different organelles, and the organelles interaction frequently implicated in cancer cells survival.

**Methods:**

Surgically excised WHO grade IV human glioblastoma tissues were collected and cells isolated for culturing. RT‐PCR and DNA sequencing assay to estimate the abundance and mutation of *Dcf1*. iTRAQ sequencing and bioinformatic analysis were performed. Subsequently, immunoprecipitation assay to evaluate the degradation of HistoneH2A isomers by UBA52 ubiquitylation. Transmission electron microscopy (TEM) was applied to observe the structure change of mitochondria and autophagosome. Organelle isolated assay to determine the distribution of protein. Cell cycle and apoptosis were evaluated by flow cytometric assays.

**Results:**

*Dcf1* was downregulated in WHO grade IV tumor without mutation, and overexpression of *Dcf1* was found to significantly regulate glioblastoma cells. One hundred and seventy‐six differentially expressed proteins were identified by iTRAQ sequencing. Furthermore, we confirmed that overexpression of *Dcf1* destabilized the structure of the nucleosome via UBA52 ubiquitination to downregulate HistoneH2A.X but not macroH2A or HistoneH2A.Z, decreased the mitochondrial DNA copy number and inhibited the mitochondrial biogenesis, thus causing mitochondrial destruction and dysfunction in order to supply cellular energy and induce mitophagy preferentially but not apoptosis. *Dcf1* also has disrupted the integrity of lysosomes to block autolysosome degradation and autophagy and to increase the release of Cathepsin B and D from lysosomes into cytosol. These proteins cleaved and activated BID to induce glioblastoma cells apoptosis.

**Conclusions:**

In this study, we demonstrated that unmutated *Dcf1* expression is negatively related to the malignancy of glioblastoma, *Dcf1* overexpression causes nucleosomes destabilization, mitochondria destruction and dysfunction to induce mitophagy preferentially, and block autophagy by impairing lysosomes to induce apoptosis in glioblastoma.

## Graphical abstract

*Dcf1* destabilized the structure of the nucleosome via downregulating HistoneH2A.X and the binding to UBA52 but not macroH2A or HistoneH2A.Z, which induced DNA damage and inhibit the recruitment of DNA repair complex. *Dcf1* overexpression decreased the mitochondrial DNA copy number, inhibited the mitochondrial biogenesis and induced mitochondrial destruction and dysfunction, thus causing the inhibition of cellular energy generation and inducing mitophagy preferentially but not apoptosis. *Dcf1* disrupt the integrality and function of lysosomes to block autophagy and induce apoptosis in glioblastoma.

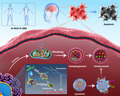

## 1 INTRODUCTION

Glioblastoma is the most common and the most lethal primary malignancy of the central nervous system (CNS), which has a high recurrence rate and mortality rate, thus, threatening human health and life worldwide severely.^1^ Currently, conventional therapies, such as surgery, radiation, and chemotherapy, and novel therapies, such as immunotherapy^2^ and nanoparticle‐based treatment methods,^3^, ^4^ have not been successful because of poor treatment outcomes and intolerable side effects. Furthermore, the key diagnostic and therapeutic targets are far being revealed due to the high heterogeneity of glioblastoma. Therefore, elucidation of the pathology and molecular biology of glioblastoma is urgently needed to improve treatment outcomes.

Thus far, many endeavors from have been made to determine the molecular mechanism of glioblastoma,^5^, ^6^, ^7^however, the heterogeneity of cellular populations and complex tumor microenvironment compositing glioblastoma tissue have hindered thoroughly exploration. Importantly, glioblastoma cells have metabolic dependencies, organellar interactions, and/or communication discrepancies that distinguish them from their normal counterparts, for example, it is known to activate or block pathways to consume more glucose and produce more lactic acid, even in normoxic conditions.^8^, ^9^ Mitochondria are powerhouses of cellular activities that are involved in mediating various biological processes of glioblastoma cells,^10^, ^11^ such as proliferation, apoptosis, multidrug resistance, signal transmission, oxidative stresses sensors, and so on. Mitochondria also act as switches to regulate tumor development^12^ and control the transformation of cellular phenotype.^13^ Although there are many sources of energy for cellular activities, the main energy process that provides energy for glioblastoma cells development and progression is still mitochondrial respiration.^14^, ^15^ In cancer cells, mitochondrial damage reduces ATP production and facilitates apoptosis or autophagy, which further accelerates lysosomal degradation to maintain cellular compartment homeostasis and mediate the survival.^16^, ^17^ Thus, mitochondria are emerging as promising targets in clinical treatments.^18^

*Dcf1*, also known as dendritic factor 1 or TMEM59, is a one‐pass transmembrane protein that has been demonstrated to play vital roles in glioblastoma cell lines in previous studies. Our group have demonstrated that *Dcf1* overexpression inhibits U251 cells through the destruction of mitochondria and the activation of apoptosis,^19^ and puried TAT‐DCF1 protein is capable to promote the apoptosis of U251 cells.^20^ More importantly, the *Dcf1* also identified to be crucial for energy balance.^21^ Thus, *Dcf1* is an important regulator of survival in glioblastoma cells. For interaction of organelles, Emilio Boada‐Romero et al demonstrated that *Dcf1* motif from amino acids 263–281 mediates the interaction of *Dcf1* with the WD‐repeat domain of ATG16L1 to promote LC3 activation and lysosomal targeting of the endosomal compartment to promote the autophagy,^22^, ^23^ and the alteration of ATG16L1 (T300A) changes the ability of the C‐terminal WD40‐repeat domain to interact with an amino acid motif that recognizes this region, then, impairing the unconventional autophagic activity with TMEM59, thus, disrupting its normal intracellular trafficking and its response to bacterial infection to increase the risk of Crohn's disease.^24^ Moreover, *Dcf1* has been identified to interact with FZD and LRP6 to form mature Wnt signalosomes that significantly to activate Wnt3a, which is responsible for regulating cell proliferation and cell fate decisions,^25^ and maintaining the stemness of neural stem cells^26^ and activation of microglia.^27^ However, the precise mechanisms of *Dcf1* in primary glioblastoma cells have not been elucidated clearly due to the high heterogeneity and complex microenvironment constitution.

In this study, we investigated the molecular mechanism of *Dcf1* in glioblastoma cells separated from surgical grade IV glioblastoma patients. In total, proteomic analysis revealed that overexpression of *Dcf1* altered the change of 176 proteins significantly and the differentially expressed proteins (DEPs) involved in the regulation of genetic materials stability and cellular energy supply. Taken deeper exploration, we found out the *Dcf1* destabilized the nucleosome structure by downregulating the expression of HistoneH2A.X but not macroH2A or HistoneH2A.Z to induce DNA damage, inhibiting the binding to UBA52 for recruiting the DNA repair complex, decreased mitochondrial DNA (mtDNA) copy number, inhibited mitochondrial biogenesis, destructed the structure and function of mitochondria, which inhibited the supply cellular energy and induced mitophagy directly and preferentially but not mitochondrial apoptosis. Moreover, *Dcf1* also led to lysosomal dysfunction and destruction, which prevented forming mature autolysosomes and blocked the process of autophagy, and increased the release of lysosomal content to cytosol, activated and executed apoptosis via cleaving BID finally (Graphical ToC). Overall, we found, for the first time, that the expression of *Dcf1* is correlated with glioblastoma malignancy and revealed the key role of *Dcf1* in the survival of human glioblastoma cells, it provides new insight for understanding glioblastoma.

## 2 MATERIALS AND METHODS

All patients in this study consented to an institutional review board‐approved protocol that allows comprehensive analysis of tumor samples (Ethics Committees of Shanghai University, PR China, the Fifth People's Hospital of Shanghai, Fudan University, PR China and Karolinska Institutet, Stockholm, Sweden.). All human glioblastoma tumor and brain tissue from trauma patients were collected at The Fifth People's Hospital of Shanghai, Fudan University, Shanghai, China. And all human glioblastoma tissue experienced curative surgery, but did not receive other therapies, and the tissue samples were confirmed as glioblastoma by a neuropathologist. This study conforms to the Declaration of Shanghai University, PR China, the Fifth People's Hospital of Shanghai, Fudan University, PR China and Karolinska Institutet, Stockholm, Sweden. All the antibodies’ information is referred to Table S1; the plasmids’ information is referred to Table S2; the primes’ information is referred to Table S3; the chemicals, kits’ information is referred to Table S4.

### 2.1 Glioblastoma cell isolation and culture

The glioblastoma tissues were separated into single cells using collagenase I for 30 min with gentle mixing every 5 min and the blood red cells were lysed with Red Blood Cell Lysis Buffer. The cells were cultured in DMEM/F12 (BI) medium with 15% FBS plus sodium pyruvate and GlutaMAX™ Supplement and 1% penicillin‐streptomycin (Life) at 37 ℃ under 5% CO_2_ in an incubator. The cell line used in this study was isolated from surgically obtained glioblastoma tissue, and no other cell lines were included.

### 2.2 Real‐time quantitative reverse transcription PCR (RT‐PCR)

Isolated tumor tissues and normal tissues were carefully dissected using surgical scissors and stored in RNA storage reagent. Total RNA was isolated using a total Eastep^®^ Super RNA extraction kit. RNA (3 μg) was reverse transcribed into cDNA using a ReverTra Ace Kit for RT‐PCR. Quantitative PCR was performed on a Rotor‐Gene Three‐Step Real‐Time PCR system. PCR was performed with SYBR^®^ Green Realtime PCR Master Mix with the following specific primers as Table S3. A total of 20 ng of cDNA or genome DNA was used per reaction. All standard curve reactions and samples were run in triplicate along with no‐template control reactions for all primer sets. The PCR cycling parameters followed the manufacturer's recommendations.

### 2.3 Cell invasion assay

A BioCoat^TM^ Matrigel^TM^ Invasion Chamber was used to assess the effect of *Dcf1* on cellular invasive ability. After transfection with pcDNA3.1 or pcDNA3.1‐Dcf1 for 48 h, cells were harvested and adjusted to a density of 5 × 10^5^/mL. After incubation in the invasion chamber for 24 h, noninvaded cells were scrubbed with a cotton swab, and invaded cells were stained with crystal violet and observed using a Nikon Ti‐S fluorescence microscope at a magnification of 100×.

### 2.4 Flow cytometry assay

After transfection with pcDNA3.1 or pcDNA3.1‐Dcf1 for 48 h, glioblastoma cells were digested with trypLE, harvested in 10% FBS in DMEM/F12, washed twice with 2% BSA in PBS, resuspended in 100 μl of binding buffer, combined with 5 μl of annexin V‐FITC and 5 μl of PI according to the protocol of an Annexin V‐FITC/PI Apoptosis Detection Kit, mixed gently and stained at room temperature for 15 min before flow cytometry detection. The cell cycle was detected by flow cytometry after digestion with 10 μg/ml RNase at 37℃ for 30 min and staining with 50 μg/ml PI at 4℃ for 30 min. The percentages of apoptotic cells and cells in each phase of the cell cycle were calculated.

### 2.5 Wound healing assay

Scratch wound healing assays were performed in 24‐well culture plates. Cells at 40%–45% confluence were transfected with pcDNA3.1 or pcDNA3.1‐Dcf1 plasmids to enhance protein expression for 24 h. Scratches were made using 1‐ml pipette tips and washed twice with culture medium. The cells were permitted to grow for an additional 48 h, fixed in 4% paraformaldehyde for 30 min and stained with 1% crystal violet in 2% ethanol for 15 min. Photographs were taken on a Nikon inverted microscope with an objective of 10×. The gap distance of the wound was measured using Image‐Pro Plus software, and the data were normalized to the data for the control group.

### 2.6 Real‐time monitoring of cellular migration assay

Real‐time monitoring of cellular migration was performed using an xCELLigence RTCA DPlus Instrument and cell invasion and migration (CIM) plates. The assays were performed after glioblastoma cells were transfected with pcDNA3.1 or pcDNA3.1‐Dcf1 for 12 h. The cells were harvested and placed into the upper chambers of CIM plates in serum‐free DMEM/F12 medium at a density of 25,000 cells per well. The bottom chambers of the CIM plates were filled with 15% serum‐containing medium to promote migration across the membrane. The CIM plates were transferred into the RTCA DP instrument, and continuous readouts were obtained for 72 h at 15‐min intervals. The experiment was performed in quadruplicate.

### 2.7 Isobaric tags for relative and absolute quantification

The isobaric tags for relative and absolute quantification (iTRAQ) were conducted with the help of Shanghai Majorbio Bio‐Pharm Technology Co., Ltd. The entire process including: (1) Protein extraction and quantification; (2) Protein digestion and iTRAQ labeling; (3) High‐pH reversed‐phase liquid chromatography (RPLC); (4) Mass spectrometry analysis; (5) Sequence database searching using Proteom Discoverer^TM^ Software 2.1 against the *Homo sapiens* database (70,611 entries); (6) Function annotation using the Blast2GO program against the nonredundant (NR) protein database (NCBI). The Kyoto Encyclopedia of Genes and Genomes (KEGG) database (http://www.genome.jp/kegg/) and the Clusters of Orthologous Groups (COG) database (http://www.ncbi.nlm.nih.gov/COG/) were used to classify and group these identified proteins.

### 2.8 Cellular ATP assay

ATP concentrations were detected using an ATP Assay Kit. In brief, cells were cultured in 6 cm dishes after transfection with pcDNA3.1 or pcDNA3.1‐Dcf1 for 48 h, lysed, and then centrifuged at 12,000 rpm for 10 min to isolate total proteins. Then, 100 μl of supernatant was added to 100 μl of ATP detection solution, and standard samples were generated. Luminescence was immediately detected using a plate reader (PerkinElmer). The protein concentration was measured using a Bradford assay.

### 2.9 Mitochondrial membrane potential assay (JC‐1 assay)

Mitochondrial membrane potential was evaluated with a JC‐1 Mitochondrial Membrane Potential Detection Kit. In brief, before staining, cells were incubated with 1:1000‐diluted CCCP as a positive control for 30 min. After transfection for 48 h, the cells were incubated with 1× JC‐1 staining solution in 24‐well plates for 20 min at 37℃ and rinsed twice with 1× staining buffer. Finally, cells cultured in 10% FBS/DMEM were detected using an LSM710 fluorescence microscope.

### 2.10 Acridine Orange staining

After transfection for 48 h, glioblastoma cells were stained with Acridine Orange (AO) at a final concentration of 5 μg/ml for 15 min (37°C, 5% CO_2_). Fluorescence images were captured using a Zeiss 710 LSM confocal microscope. AO produces red fluorescence (with an emission peak at approximately 650 nm) in lysosomal compartments and green fluorescence (with an emission peak between 530 and 550 nm) in cytosolic and nuclear compartments. The fluorescence intensity per pixel was quantified with Image‐Pro Plus software, and the ratio of red and green fluorescence intensity per pixel was calculated.

### 2.11 Transmission electron microscope

Primary glioblastoma cells transfected with pcDNA3.1 or pcDNA3.1‐Dcf1 were cultured in complete medium for 48 h, and then, the cells were fixed in 2% formaldehyde and 2.5% glutaraldehyde. The fixed samples were postfixed with osmium tetroxide, dehydrated, and embedded in Araldite Durcupan ACM. Then, ultrathin sectioning was conducted, and the sections were photographed with a transmission electron microscope.

### 2.12 Isolation of the lysosomal fraction

Cells transfected with pcDNA3.1 or pcDNA3.1‐Dcf1 for 48 h were washed in precooled PBS, trypsinized, quenched with medium, and centrifuged at 500× *g* for 3 min to produce cell pellets. After medium removal, the pellets were washed with precooled PBS and centrifuged at 500× *g* for 3 min again. The primary glioblastoma cell pellets were resuspended in sucrose homogenization buffer (0.25 M sucrose, 20 mM HEPES) containing PMSF, protease inhibitor, and phosphatase inhibitor; homogenized by sonication; and ground. The homogenates were centrifuged at 500× *g* for 5 min, and the unbroken cell debris was discarded. The resulting supernatant (S1) was centrifuged at 6800 g for 10 min to yield P2 (mitochondrial fraction). The remaining supernatant (S2) was centrifuged again at 20,000× *g* for 40 min to yield P3 (the lysosomal fraction), which was washed with fresh sucrose homogenization buffer and centrifuged at 20,000× *g* for 15 min. The P2 and P3 fractions were resuspended in RIPA buffer containing protease and phosphatase inhibitors for further detection.

### 2.13 Labeling of mitochondria/lysosomes

Mitochondria and lysosomes were labeled with MitoTracker Green and LysoTracker Red, respectively, according to the manufacturer's protocol. Briefly, the labeling reagent was added to cultured cells at a 1:1,000 dilution, and the cells were incubated at 37°C for 30 min. Images were obtained using a fluorescence microscope.

### 2.14 Lysosomal acid phosphatase (ACP) assay

ACP activity was assayed with a commercially available kit according to the manufacturer's instructions. Glioblastoma cells transfected with pcDNA3.1‐Dcf1 or pcDNA3.1 were collected, lysed and centrifuged at 10,000 rpm at 4℃ for 5 min, and the supernatant was removed and placed in a new tube for testing. After incubation with detection reagent, the absorbance was recorded at 450 nm using a microplate reader.

### 2.15 Immunofluorescence (IF)

Glioblastoma cells were seeded, transfected for 48 h, rinsed with PBS, fixed in 4% paraformaldehyde for 15 min at room temperature, permeabilized with 0.1% Triton X‐100 for 15 min, blocked with 5% BSA‐PBS at room temperature for 90 min, and incubated with primary antibodies at 4°C overnight. After they were washed with PBS, the cells were incubated with secondary antibodies for 2 h at room temperature, and fluorescence was obtained using an LSM710 fluorescence microscope.

### 2.16 Immunoprecipitation assay

Glioblastoma cells were seeded in 10‐cm culture dishes, transfected with pcDNA3.1 or pcDNA3.1‐Dcf1 for 48 h, rinsed with PBS three times and collected in to 10 ml centrifuge tube, and lysed with cell lysis buffer contain protein inhibitors. The lysate was incubated with the protein A/G beads that coupled with UBA52 antibody 4°C overnight. Then, the protein A/G beads were collected via centrifuging at 3000 rpm for 5 min and washed with PBS three times, the desorption of bound protein were treated with glycine (0.1 M, pH2.5), boiled and examined with western blotting.

### 2.17 Western blotting analysis

The protein abundance values of the pooled samples previously analyzed by iTRAQ LC‐MS/MS were confirmed. Protein samples (35 μg) were added to electrophoretic buffer containing β‐mercaptoethanol prior to SDS‐PAGE and transferred onto 0.45 or 0.22 μm nitrocellulose membranes. The membranes were blocked with 5% milk in PBS for 1.5 h and subsequently probed by using the following primary‐specific antibodies (as shown in Table S1). Tubulin and GAPDH were used as loading controls. After the samples were incubated with affinity‐purified goat anti‐rabbit DyLight 800‐conjugated or goat anti‐mouse DyLight 680‐conjugated secondary antibodies (1:10,000) for 1.5 h, images were acquired by using an Odyssey infrared imaging system at 700 and 800 nm in the 16‐bit TIFF format. Odyssey software was applied for the quantification of protein expression.

### 2.18 Statistical analysis

For cell‐based assays, independent experiments were performed. The results of Western blotting analyses of differential protein expression were validated in cell lysates from three biological replicates. Statistical significance was analyzed using Student's *t*‐test or ANOVA by using GraphPad Prism v7.01 software (GraphPad Software). Statistical significance is expressed as *p < 0.05, **p < 0.01, ***p < 0.001, and ****p < 0.0001.

## 3 RESULTS

### 3.1 *Dcf1* expression is decreased with tumor malignancy but lacks mutation

To thoroughly investigate the relationship between *Dcf1* and glioblastoma, 24 WHO grade IV surgical glioblastoma tissues were collected, and 11 glioblastoma cell lines were isolated for culture successfully. First, the expression of *Dcf1* was explored, and the results showed that *Dcf1* was downregulated in glioblastoma tissue compared to normal tissue in mRNA and protein (Figure S1A,B), it is consent with data from the gene microarray database (GDS2853, GSE12305; Figure S1C), which implies that the expression level is negatively related to tumor malignancy. Moreover, we explored the mutation by DNA sequencing, the results showed that there is no mutation in full length of *Dcf1* gene (Figure S1D), which is not similar with the oncogenes or antioncogenes, such P53, KRAS, and PTEN in glioblastoma.

### 3.2 *Dcf1* destabilizes the stability of genetic material

**FIGURE 1:**
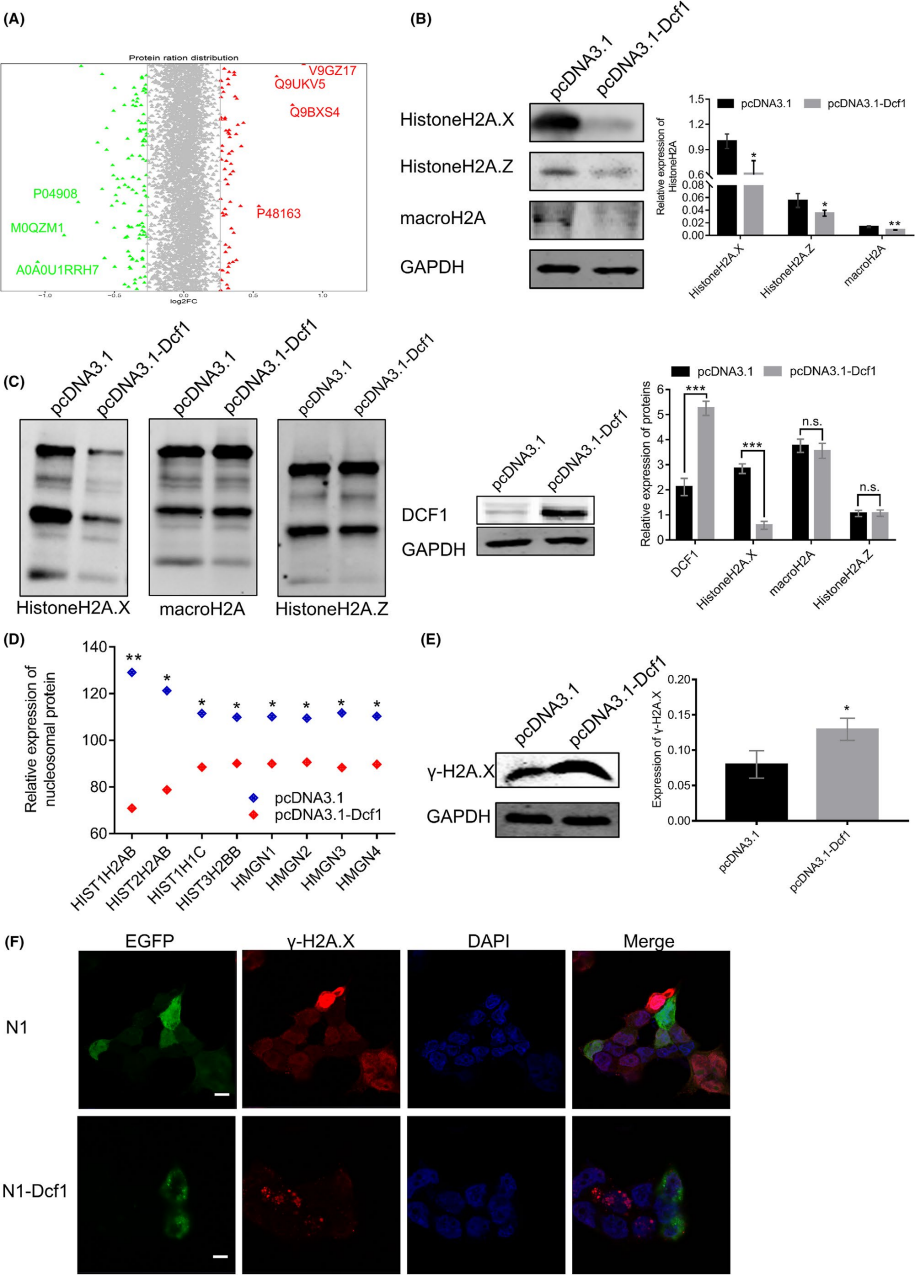
Dcf1 destabilized the structure of nucleosomes and damaged DNA. (A) Scatter plot of the protein expression (*n* = 3). Red: upregulated proteins; green: downregulated proteins; gray: unchanged proteins. (B) Detection of HistoneH2A isomer expression using Western blotting (*n* = 3). (C) Immunoprecipitation of UBA52 and HistoneH2A isomers. (D) Summary of nucleosome‐related protein changes determined by iTRAQ (*n* = 3). (E) Evaluation of DNA damage with γ‐H2A.X (*n* = 3). (F) Immunofluorescence image of γ‐H2A.X. Scale bar: 50 μm. Data were presented as mean ± SEM. Significance between every two groups was calculated by the Student's *t*‐test. *p < 0.05, **p < 0.01, ***p < 0.001

In previous studies, *Dcf1* promoted apoptosis in U251 cell.^19^, ^20^ Hence, we applied iTRAQ‐based proteomics approach to obtain a comprehensive understanding profile of the molecular mechanisms in primary glioblastoma cells. A total of 176 DEPs were identified after *Dcf1* overexpression (Figure 1A; 64 were upregulated, Table 1), 112 were downregulated (Table 2), and the differential expression was confirmed by western blotting (Figure S2A,B). Given the results of deeper bioinformatic analysis combined with the results of previous study,^28^, ^29^ the DEPs mainly involved in regulating the stability of genetic material and cellular energy supply (Figure S2C,D), both are crucial for the cell survival. As expected, *Dcf1* induced the most significant decrease in HistoneH2A (Accession: A0A0U1RRH7, Figure 1A), the core component of nucleosomes and a modulator that stabilizes DNA to control different biological processes. However, *Dcf1* did not alter HistoneH2B, HistoneH3, HistoneH4 via iTRAQ sequencing data; thus, we assumed that *Dcf1* induces primary glioblastoma cells into apoptosis via altering the stability of genetic material. And the bioinformatic analysis revealed that the *Dcf1* leaded to the reduction of UBA52 (Figure S2E), a ribosomal protein, which links to proteins and is responsive for the degradation of protein and the recruitment of the DNA damage repair complex.^30^, ^31^ As shown in Figure 1B, the western blotting results showed that *Dcf1* induced the downregulation of three isomers of HistoneH2A significantly, and the immunoprecipitation results revealed that only HistoneH2A.X was decreased linked to UBA52 (Figure 1C), thus, *Dcf1* inhibits the recruitment of the DNA damage repair complex. In addition, the bioinformatic analysis results confirmed that the expression of nucleosome core‐related proteins, including HIST1H2AB, HIST2H2AB, HIST1H1C, and HIST3H2BB (Figure 1D), was also decreased significantly, which prevented DNA histone assembly and thus prevented formation of nucleosome and condensation to form chromosomes. Moreover, *Dcf1* also induced the decrease of HMGN1, HMGN3, and HMGN4 (Figure 1D), exposing transcription activation/inactivation regions to cause DNA damage (Figure 1E,F) that affected the stability of genetic material and induced apoptosis,^32^ which is similar to our group's research in U251 cells.^29^

**TABLE 1:** List of selected differentially upregulated expressed proteins in glioblastoma cells

Rank#	Accession	Gene symbol	FC (DCF1/EGFP)	Unique peptides	AA	MW (kDa)
1	V9GZ17	TUBA8	1.82	1	275	31.051
2	Q9H993	ARMT1	1.292	3	441	51.14
3	Q8NHH9	QTL2	1.299	3	583	66.187
4	Q969Y2	GTPBP3	1.31	1	432	52.026
5	O00401	WASL	1.3	2	505	54.793
6	P48163	ME1	1.455	1	572	64.109
7	Q9UKV5	AMFR	1.59	1	643	72.949
8	Q96CW5	TUBGCP3	1.347	1	907	103.506
9	Q9UNI6	DUSP12	1.287	1	340	37.663
10	Q7Z4G4	TRMT11	1.333	1	463	53.387
11	P07948	LYN	1.283	1	512	58.537
12	Q9Y2H2	INPP5F	1.286	2	1132	128.326
13	P48201	ATP5G3	1.328	1	142	14.684
14	Q9H4I9	SMDT1	1.303	1	107	11.434
15	M0QYN0	MYDGF	1.278	1	189	20.375
16	Q8TD26	CHD6	1.248	1	2715	305.22

Note

FC: expression fold change, the abundance ratio of DCF1 and EGFP. AA: the number of amino acid. MW: molecular weight of selected protein.

**TABLE 2:** List of selected differentially downregulated expressed proteins in glioblastoma cells

Rank#	Accession	Gene symbol	FC (DCF1/EGFP)	Unique peptides	AA	MW (kDa)
1	M0QZM1	HNRNPM	0.482	1	383	40.016
2	P62987	UAB52	0.665	1	128	14.719
3	A0A0U1RRH7	Histone H2A	0.384	1	170	18.541
4	P04908	HISTAH2AB	0.55	1	130	14.127
5	Q15642	TRIP10	0.701	3	601	68.31
6	Q8IUE6	HIST2H2AB	0.65	1	130	13.987
7	Q13480	GAB1	0.703	1	694	76.569
8	P50552	VASP	0.59	1	380	39.805
9	Q6UXH1	CRELD2	0.68	2	353	38.166
10	Q9UNZ5	C19orf53	0.665	2	99	10.57
11	P48509	CD151	0.698	2	253	28.276
12	P20933	AGA	0.648	1	346	37.184
13	O14734	ACOT8	0.673	1	319	35.891
14	Q9Y639	NPTN	0.599	1	398	44.36
15	Q92551	IP6K1	0.68	1	441	50.204
16	P16104	H2AFX	0.758	5	143	15.135

Note

FC: expression fold change, the abundance ratio of DCF1 and EGFP. AA: the number of amino acid. MW: molecular weight of selected protein.

### 3.3 *Dcf1* causes mitochondrial destruction and dysfunction to induce mitophagy

**FIGURE 2:**
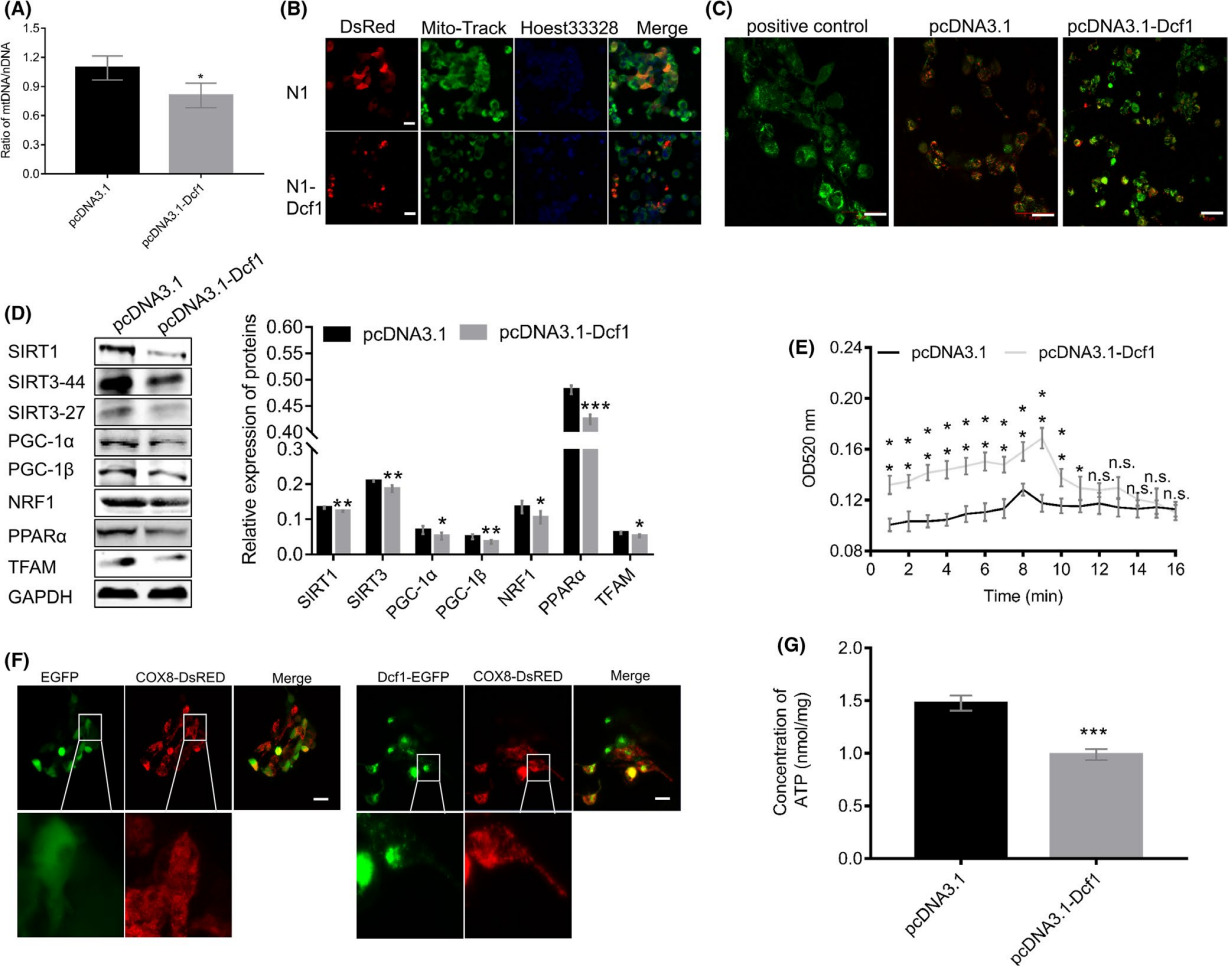
Dcf1 destroyed mitochondria. (A) The mtDNA/nDNA ratio detected with RT‐PCR (*n* = 6). (B) Mitochondrial staining with MitoTracker Green. (C) Detection of mitochondrial membrane potential with a JC‐1 kit. (D) Western blotting results for the mitochondrial biogenesis pathway (*n* = 3). (E) Detection of isolated mitochondrial membrane permeability transition pores (MPTPs) with Ca^2+^ absorbance examination. (F) Immunofluorescence images of mitochondrial structure. (G) ATP concentrations in glioblastoma cells determined using an ATP Assay Kit (*n* = 4). Scale bars: 50 μm. Data were presented as mean ± SEM. Significance between every two groups was calculated by the Student's *t*‐test. *p < 0.05, **p < 0.01, ***p < 0.001

The previous results showed that *Dcf1* destabilized genetic material, and the crucial regulation of genetic materials in cell survival and fate. On the other hand, the *Dcf1* has been identified to express at mitochondria and induces the loss of mitochondrial localization of MGST1.^28^ However, the precise molecular mechanism of *Dcf1* between genetic materials and cellular survival is still unknown. Hence, we conducted deeper bioinformatic analysis on the iTRAQ sequencing data. The results showed that the localization of DEPs in mitochondria and lysosomes was opposite that in the remaining organelles (Figure S3A,B), which suggested that the *Dcf1* controls the viability of glioblastoma cell via the structure and function of mitochondria and lysosomes. As we all known, the status of mitochondrial DNA (mtDNA) and mitochondrial physiology were controlled by nucleus DNA (nDNA), and mitochondria and lysosome are critical for cell death and apoptosis.^19^, ^33^, ^34^ Therefore, we explored the relationships of *Dcf1* with mitochondria and inter‐organelle interactions in glioblastoma cells. First, we observed that *Dcf1* decreased the ratio of mtDNA to nDNA (Figure 2A), which could alter the physiology and function of mitochondria, including cellular respiration. Mito‐Tracker Green staining revealed that *Dcf1* decreased the number of mitochondria (Figure 2B) and inhibited the biogenesis of mitochondria via the SIRT1/SIRT3‐PGC1α‐NRF1‐TFAM pathway (Figure 2C).^35^, ^36^ More, the mitochondrial membrane potential examination conclusively showed that *Dcf1* reduced the membrane potential (Figure 2D) and increased the permeability of the mitochondria membrane via the permeability transition pore (PTP; Figure 2E), these can lead to decreased release (efflux) or increased uptake (influx) of ions and materials by above destruction synergistically, such as Ca^2+^ and K^+^. Immunofluorescence with COX8‐DsRed revealed mitochondrial fragmentation upon ectopic *Dcf1* expression (Figure 2F), implying *Dcf1* has a destructive effect on mitochondrial structure and induced the dysfunction of mitochondria. As expected, the ATP concentrations in glioblastoma cells were significantly reduced (Figure 2G). The results of further examination also showed that the content of intracellular Ca^2+^ was elevated (Figure S3C), suggesting the existence of mitochondrial dysfunction and communication improper between organelles that impacted cell viability.

**FIGURE 3:**
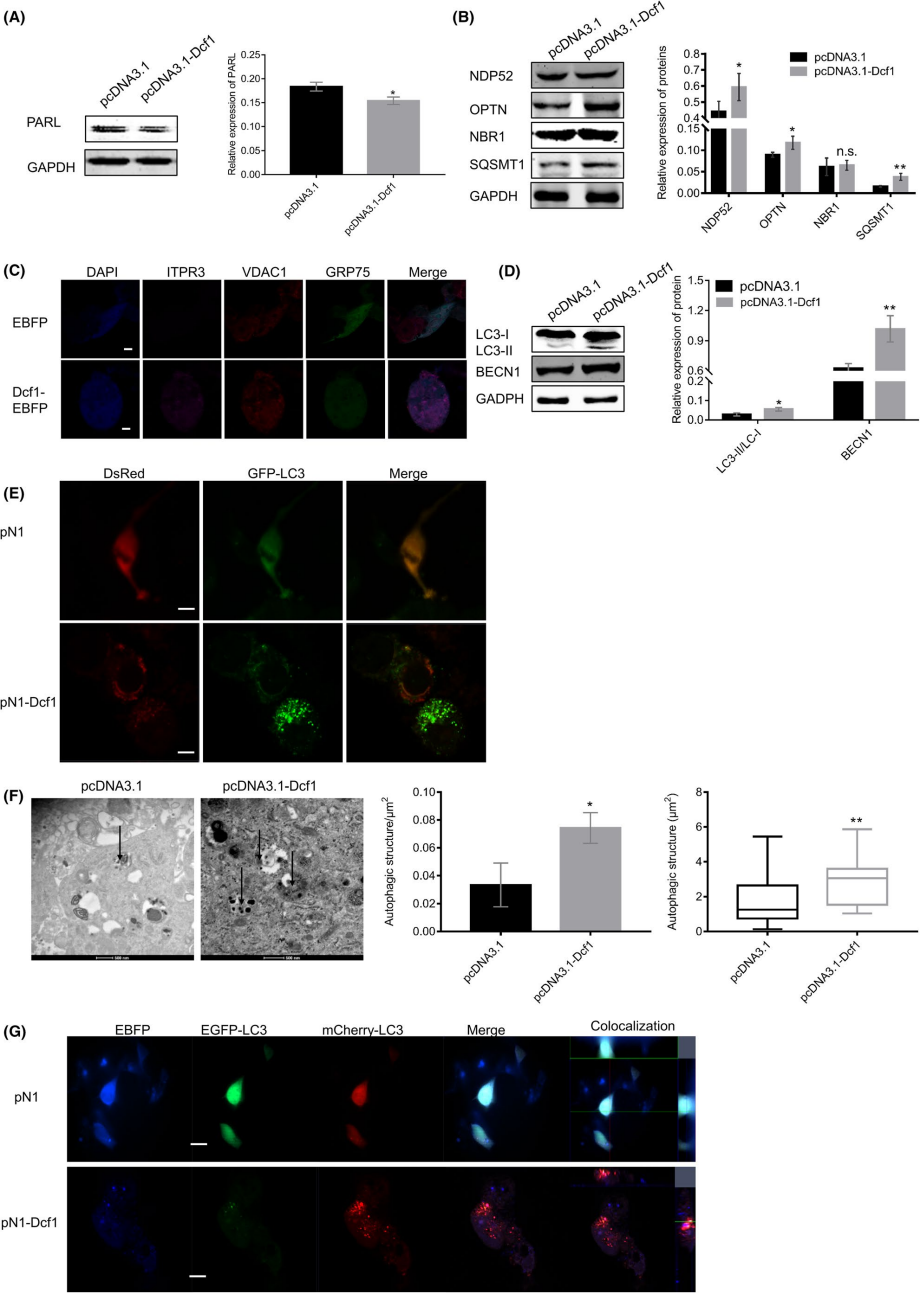
Dcf1 activated mitophagy in glioblastoma cellss. (A) PARL expression determined by Western blotting (*n* = 4). (B) Western blotting results for mitophagy receptors (*n* = 4). (C) Immunofluorescence image of mitophagy. (D) Western blotting of LC3‐II/LC3‐I (*n* = 4). (E) Representative images of GFP‐LC3 puncta in glioblastoma cells. (F) Ultrastructural evidence showing elevated autophagy levels in glioblastoma cells. (G) Dcf1 promoted the fusion of APs and lysosomes. Scale bars: 50 μm. Data were presented as mean ± SEM. Significance between every two groups was calculated by the Student's *t*‐test. *p < 0.05, **p < 0.01

Mitochondria play important roles in cell survival and the response of stress different stimulus, damaged mitochondria can induce autophagy or apoptosis via the mitochondrial pathway,^37^ however, the processes of apoptosis and autophagy in glioblastoma cells are interdependent and antagonistic. A previous study has reported that the disordered autophagy and apoptosis favor to the genesis and development of tumors; thus, autophagy and apoptosis are essential to cancer survival. These processes are regulated by PARL^38^ and BECN1/Bcl‐2.^39^, ^40^ Western blotting showed that *Dcf1* inhibited the expression of PARL (Figure 3A), and the subcellular colocalization of BECN1‐Bcl‐2 in endoplasmic reticulum (ER) was not changed by *Dcf1* (Figure S4A), suggesting that autophagy and apoptosis occurred in glioblastoma cells simultaneously. However, *Dcf1* increased BECN1/Bcl‐2 ratio after 24 h, which suggested that *Dcf1* preferentially drove glioblastoma cells to undergo autophagy (Figure S4B).

Then, mitophagy receptors, such as NDP52 (also known as CALCOCO2), NBR1, OPTN, and SQSTM1,^41^ were examined as indexes of mitophagy. As shown in Figure 3B, NDP52, OPTN, and SQSTM1 were significantly increased but NBR1 was not changed, as well as the other results of immunofluorescence and western blotting with VDAC1‐GRP75‐ITPR3 complex (Figure 3C; Figure S4C). Directly, the results showed that *Dcf1* significantly increased the levels of the autophagic markers LC3‐II/LC3‐I and BECN1 (Figure 3D). Furthermore, autophagy was validated performed by immunofluorescence and transmission electron microscopy (TEM). The signals of autophagosome (AP)‐associated LC3 (GFP puncta) were significantly increased and AP puncta were less able to form in the absence of *Dcf1* overexpression (Figure 3E). TEM directly confirmed the increase in the number of Aps (Figure 3F). Interestingly, we also observed that the APs were larger in the *Dcf1* overexpression group than in the control group (Figure 3F). In addition, the colocalization of GFP‐LC3 and mCherry‐LC3 was observed with pN1‐DsRed, *Dcf1* promoted mCherry‐LC3 colocalization, but few GFP‐LC3 puncta between GFP‐LC3 and mCherry‐LC3, suggesting the existence of functional autolysosomes and the fusion of APs with lysosomes (Figure 3G). Meanwhile, the mitochondrial destruction and dysfunction with *Dcf1* to induce mitophagy via the PI3K/Akt/MAPK/mTOR/Ulk1 pathway (Figure 3D).^42^, ^43^

### 3.4 *Dcf1* disrupts the integrality of lysosomes and blocks autophagy

**FIGURE 4:**
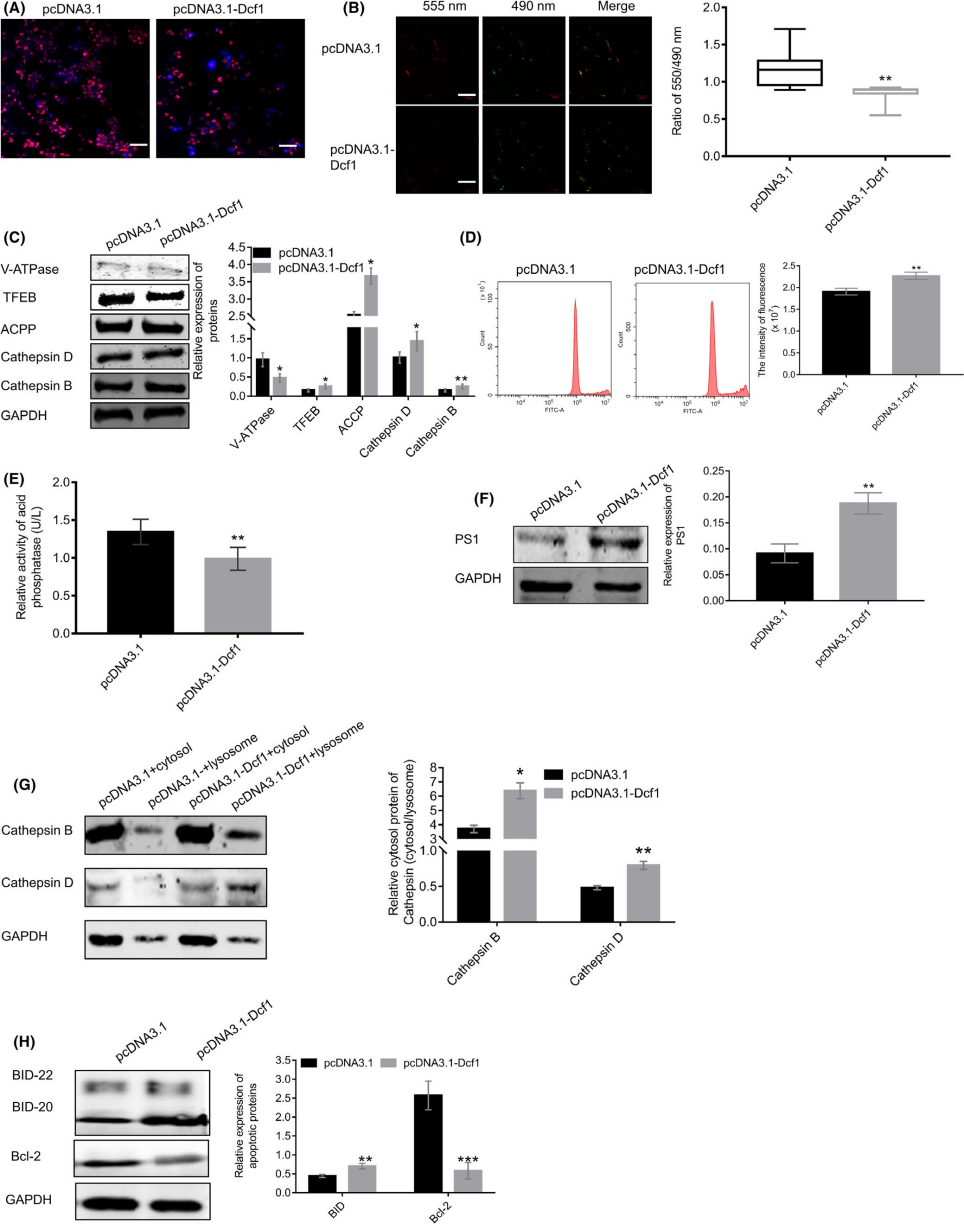
Dcf1 disrupted the integrity of lysosomes and blocked the process of autophagy. (A) Representative images of LysoTracker Red staining. (B) Representative images and summary of the results of lysosome staining with acridine orange. (C) Western blotting detection of lysosomal proteins (*n* = 4). (D) Image of pH determination via flow cytometry. (E) Detection of acid phosphatase activity with an acid phosphatase kit (*n* = 4). (F) Western blotting detection of PS1 (*n* = 4). (G) Western blotting detection of Cathepsin B and Cathepsin D release from lysosomes into the cytosol (*n* = 3). (H) Western blotting detection of cleaved BID and Bcl‐2 (*n* = 4). Scale bars: 50 μm. Data were presented as mean ± SEM. Significance between every two groups was calculated by the Student's *t*‐test. *p < 0.05, **p < 0.01, ***p < 0.001

**FIGURE 5:**
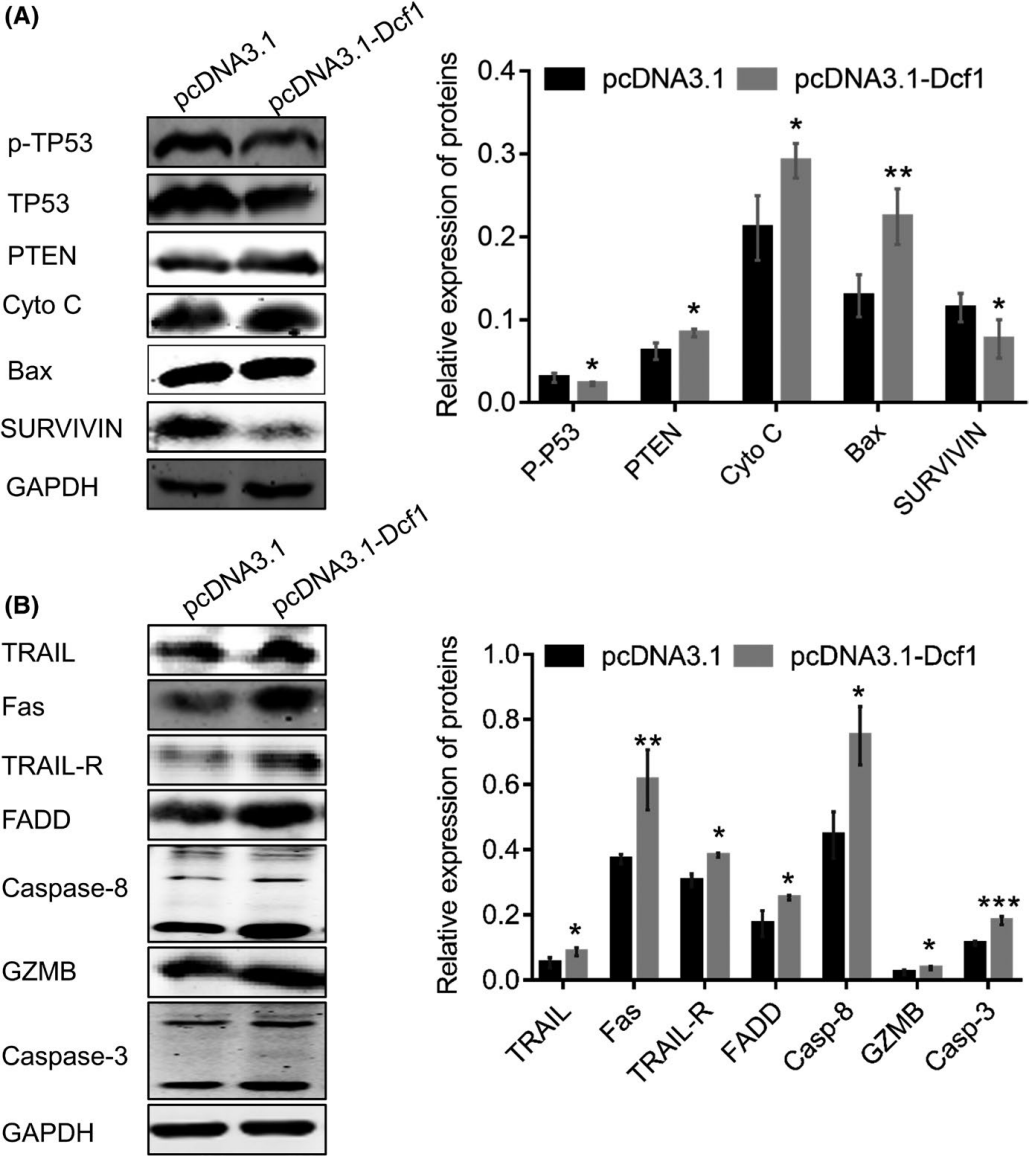
Dcf1 regulated apoptosis of glioblastoma cells via the extrinsic death receptor apoptotic pathway. (A) Western blotting detection of apoptosis‐related proteins (*n* = 4). (B) Western blotting detection of the extrinsic death receptor apoptosis pathway (*n* = 4). Data were presented as mean ± SEM. Significance between every two groups was calculated by the Student's *t*‐test. *p < 0.05, **p < 0.01, ***p < 0.001

Intact autophagy involves the fusion of APs with lysosomes to form mature autolysosomes that the cargo from autophagic vesicles were degraded via lysosomal enzymes, and autophagy malfunction can directly affect cancer cell survival.^44^, ^45^ We observed that *Dcf1* significantly decreased the number of lysosomes (Figure 4A), disrupted the integrality of lysosomes and obviously enhancing the permeability of the lysosomal membrane (causing lysosomal membrane permeabilization, LMP; Figure 4B). For example, V‐ATPase and TFEB were downregulated, and the lysosomal inclusions Cathepsin D and Cathepsin B were upregulated (Figure 4C). The reduction V‐ATPase led to the failure of establishment and maintenance of the lysosomal lumen pH at approximately 4.6 to keep the activity of most lysosomal hydrolases,^46^, ^47^ the inhibition of TFEB represses the biogenesis of lysosomes and increases the formation of Aps.^48^, ^49^ The changes in lysosomal structure and constitution induced the formation to dysfunctional lysosomes, sequentially, inhibiting the activity of hydrolases, increasing the release of lysosomal components into cytosol and changing lysosomal inclusions. As expected, the lysosome pH was basic (Figure 4D) and the activity of acid phosphatase was inhibited (Figure 5E), the increases in lysosomal pH and decreases in acid phosphatase activity lead to dysfunction of hydrolysis, which hinders degradation and trafficking.^50^ Besides, the western blotting results of Rab5 and Rab7 in tumor tissue were decreased, it implied that the hydrolases within lysosomes from endosomes were reduced and the proper function of lysosome was hindered (Figure S5A); however, *Dcf1* significantly increased the expression of EEA1, Rab5, and Rab7 (Figure S5B), suggesting that the sufficient supply of hydrolases to lysosomes from endosomes. Given these finding, we concluded that *Dcf1* disrupts the integrity of lysosomes in glioblastoma cells. Moreover, *Dcf1* also promoted the fusion of lysosome to APs via enhances the expression of presenilin 1 (PS1) (Figure 4F).^51^ However, *Dcf1* suppressed lysosome‐associated membrane protein 1 (LAMP1) expression significantly and induced the aggregation obviously (Figure S5B,C), which hindered the formation of autolysosomes.^52^, ^53^ Due to the change of lysosome membrane protein and the integrity with *Dcf1*, accelerated the release of inclusion in the lysosomal lumen to the cytosol, here, we observed that the level of lysosomal aspartyl protease, Cathepsin D, acid phosphatase, Cathepsin B in cytoplasm was increased after isolated the lysosome from glioblastoma cells (Figure 5G; Figure S5D,E, indicated by the arrow).

Then, we observed that the Cathepsin B and Cathepsin D in cytosol promoted the cleavage and activation of BID (Figure 4H), which could promote the mitochondrial release of proapoptotic factors into the cytosol to trigger apoptosis sequentially. And the reduction in Bcl‐2 (Figure 4F) resulted to the opening of the mitochondrial voltage‐dependent anion channel (VDAC), which leaded to the loss of membrane potential and the release of cytochrome C into the cytosol to execute the process of apoptosis.^54^

### 3.5 *Dcf1* inhibits the viability and promotes glioblastoma cells apoptosis

We have proved that the enhanced expression of *Dcf1* destabilized the genetic materials, resulted to DNA damage, and caused mitochondria to inhibit the ATP generation and induced mitophagy, then also caused the damage to lysosome that blocked the process of autophagy and increased the release of lysosomal inclusion into cytosol, cleaved and activated BID. Hence, we explored the viability of glioblastoma cells with *Dcf1*. First, we observed that *Dcf1* significantly reduced the proliferation rate and migratory capacity of glioblastoma cells (Figure S6A), which are critical characteristics for cell mobility and absorbability (Figure S6B,C). In addition, *Dcf1* significantly inhibited the invasion and metastasis of glioblastoma cells (Figure S6D,E). On the other hand, *Dcf1* failed to arrest the cell cycle (Figure S6F) but clearly induced glioblastoma cells to undergo apoptosis (Figure S6G,H). These results reveal that overexpression of *Dcf1* is crucial for the regulation of the viability of glioblastoma.

Besides, we wondered that whether *Dcf1* was beneficial for inducing glioblastoma cells to undergo cellular senescence and cellular death, because of the decreased expression of HistoneH2A and destabilization of chromatin to affected the transcription and translation within the glioblastoma cells.^55^ Surprisingly, *Dcf1* was not capable of promoting the glioblastoma cell senescence (Figure S7A,B). Meanwhile, we observed that *Dcf1* enhanced the expression of proapoptotic factors and inhibited the expression of antiapoptotic factors; for example, it decreased the phosphorylation of TP53; enhanced the expression of PTEN, Cyto C and Bax; and inhibited the expression of antiapoptotic proteins survivin and Bcl‐2 (Figures 4G and 5A). The decreases of survivin and Bcl‐2 favored direct activation of caspase‐3/‐7, bound to pro‐caspase‐9, and promoted the recruitment of Apaf‐1 to promote apoptosis. Thus, these decreases drove glioblastoma cells to undergo apoptosis. Then, we identified that the destruction and dysfunction of mitochondria and the blockade of autophagy regulated glioblastoma cells apoptosis via the extrinsic death receptor apoptosis signaling pathway. The levels of TRAIL, Fas and their corresponding receptor and PDGF, PDGFR, and TRAIL, TRAIL‐R, Fas, PDGF‐A/B and TRADD showed significant activation; however, PDGFR‐β was downregulated. These results suggested that *Dcf1* induced apoptosis in glioblastoma cells via the extrinsic death receptor apoptosis signaling pathway (Figure 5B; Figure S8). Downstream of this pathway, TRAIL‐R, Fas and TNF‐α‐R1 bound to the cytosolic Fas‐associated death domain adaptor protein (FADD; Figure 5B), and the apoptosis initiators procaspase‐8 and/or procaspase‐10 were bound to activated FADD, completing the formation of death‐inducing signaling complexes (DISCs). Within the DISCs, procaspase‐8 and procaspase‐10 were activated via homodimerization and cleaved and activated procaspase‐3 and procaspase‐7 into active caspase‐3, caspase‐7 and the granzyme‐B (GZMB), eventually resulting in apoptosis of the target cells. Caspase‐3, caspase‐7, and GZMB are the executors of apoptosis and induce change in tubulin, Mcl‐1, Lamin A/C, PARP and ICAD (Figure S8). Increase of tubulin enhanced the formation of nuclear complexes between tubulin and TUBGCP3 to promote the effects of the CDK5 regulatory subunit‐associated tumor suppressor protein 3 (TP53) on DNA damage in glioblastoma cells,^56^ which work synergistically to induce the apoptosis of glioblastoma cells. Collectively, the finding indicated that *Dcf1* blocked the process of autophagy and broke down the cellular homeostasis to induce cells into apoptosis.

## 4 DISCUSSION

Despite the myriad of studies that have focused on elucidating the molecular mechanisms of glioblastoma with available technologies from various perspectives,^57^, ^58^, ^59^ the mechanisms are still far from clear, and the results of treatment have been disappointed. In this study, we collected a cohort of glioblastoma tissues, combining cases from published gene microarray datasets. We found that *Dcf1* can affect the viability of glioblastoma cells in various ways, such as by inhibiting invasion, migration, and proliferation and inducing glioblastoma cell apoptosis. We applied an iTRAQ‐based proteomics approach to explore further molecular mechanisms. Bioinformatics analysis revealed that DEPs occurred in critical molecular and signal pathways that regulate different biological processes or organelle interactions. We observed that *Dcf1* destabilized genetic material by decreasing the expression of HistoneH2A.X to affect the structure of the nucleosome and inhibiting the binding of HistoneH2A.X with UBA52 to recruit the DNA repair complex, which induced the destabilization of genetic materials and a decrease in mtDNA copy number and inhibited the biogenesis of mitochondria via the SIRT1/SIRT3‐PGC1α‐NRF1‐TFAM pathway. As a result, *Dcf1* induced destruction of the glioblastoma cell mitochondrial structure, resulting in mitochondrial dysfunction that hindered ATP production. The mitochondrial damage caused glioblastoma cells to undergo autophagy preferentially by downregulating the expression of PARL and changing the ratio of BECN1/Bcl‐2 and the process of mitophagy was regulated by the PI3K/Akt/MAPK/mTOR/Ulk1 pathway. However, *Dcf1* also destroyed the integrity of the lysosomal membrane by increasing LAMP1 expression and changed the constitution of lysosomal membrane proteins. Appropriate function was therefore unable to be maintained in the lysosomal lumen, which inhibited the degradation of autolysosomes and increased the release of Cathepsin B and Cathepsin D into the cytosol lumen. Cathepsin B and Cathepsin D release into the cytosol promoted the cleavage and activation of BID, and *Dcf1* also decreased the expression of Bcl‐2 to induce the release of proapoptotic factors and enhance the levels of cancer suppressors. Finally, *Dcf1* induced apoptosis via extrinsic the death receptor apoptosis signaling pathway and caused the glioblastoma cell processed toward death.

Previous studies have demonstrated that autophagy and apoptosis collaborate to control the survival and development of cancer cells.^16^, ^60^ Where and when autophagy or apoptosis arises or dominates in different cancer,the subtle changes in interactions between different organelles, such as interactions of mitochondria associated membranes (MAMs) with the ER and lysosomes that respond to endogenous or exogenous changes; and the precise pathway that controls apoptosis or autophagy is poorly understood. Here, we observed that *Dcf1* preferentially induced glioblastoma cells to undergo apoptosis via autophagy. However, we could not conclude which change occurred first or whether they were simultaneous in this study and need further exploration to be conducted.

While this work was in progress, we became aware of another study on the complex signal network,^58^ but the tumor microenvironment was markedly different from those of tissue‐derived glioblastoma cells, which might have important effect on the experimental results compared to cell lines. It is obvious that further improvements in culture and transplantation conditions (possibly through many efforts to mimic real‐life environments) will be essential steps for enhanced understanding and potential clinical development. Nevertheless, our study provides insights into the complex regulation that occurs during glioblastoma, in which host defenses system and other processes, such as the mitochondrial metabolism, are switched on/off.^61^, ^62^

Eradication of tumors with appropriate external stimuli might be a better alternative therapeutic strategy for patients in whom standard therapies have failed. Although the targets of glioblastoma are uncertain, our study established that *Dcf1* is a potential suppressor and candidate target for glioblastoma treatment with both genetic and biological evidence, providing new information with which to understand mitochondrial function, autophagy, and lysosomal function in glioblastoma pathology.

## 5 CONCLUSION

In summary, primary glioblastoma cells were isolated from grade IV human glioblastoma tissues. We found that *Dcf1* was downregulated without mutation, and overexpression of *Dcf1* inhibited the viability of glioblastoma cells significantly. We identified that the overexpression of *Dcf1* has destructive effects on genetic materials stability and inhibitive effects on cellular energy supply with iTRAQ sequencing. *Dcf1* destabilized the structure of the nucleosome via downregulating HistoneH2A.X and the binding to UBA52 but not macroH2A or HistoneH2A.Z, which induced DNA damage and inhibit the recruitment of DNA repair complex, decreased the mitochondrial DNA copy number, inhibited the mitochondrial biogenesis and induced mitochondrial destruction and dysfunction, thus causing the inhibition of cellular energy generation and inducing mitophagy preferentially but not apoptosis. Meanwhile, *Dcf1* disrupted the integrity of lysosomes to block autolysosome degradation and autophagy and to increase the release of lysosomal content into cytosol, these contributed to inhibit the survival of glioblastoma via increasing the apoptosis (Graphical abstract).

## AUTHORS' CONTRIBUTIONS

G.L. conceptualized the project, isolated and cultured the primary glioblastoma cell, carried out the cell viability detection, TEM imaging, western blotting, iTRAQ analysis, data collection and wrote the manuscript. R.F., analyzed experimental results. W.L., carried out the immunofluorescence experiments. Y.C. carried out the experiments of the structure and function of mitochondria. Y.S. carried out co‐immunoprecipitation experiments. J.M conceptualized the project, iTRAQ analysis and revised the manuscript. Y.D. conceptualized the project and revised the manuscript. T.W. conceptualized the project and revised the manuscript. All authors have read and approved the manuscript.

## ETHICS APPROVAL AND CONSENT TO PARTICIPATE

All patients consented to an institutional review board‐approved protocol that allows comprehensive analysis of tumor samples (Ethics Committees of Shanghai University, PR China, the Fifth People's Hospital of Shanghai, Fudan University, PR China and Karolinska Institutet, Stockholm, Sweden.). This study conforms to the Declaration of Shanghai University, PR China, the Fifth People's Hospital of Shanghai, Fudan University, PR China and Karolinska Institutet, Stockholm, Sweden. And no potentially identifiable human images or data is presented in this study.

## CONSENT FOR PUBLICATION

Not applicable.

## COMPLIANCE AND ETHICS

The authors declare that they have no competing interests.

## Supporting information

Supplementary Material

## Data Availability

All data generated or analyzed during this study are included in this published article.

## References

[1] Siegel RL , Miller KD , Fuchs HE , Jemal A . Cancer Statistics, 2021. CA Cancer J Clin. 2021;71:7‐33. 33433946 10.3322/caac.21654

[2] Lim M , Xia YX , Bettegowda C , Weller M . Current state of immunotherapy for glioblastoma. Nat Rev Clin Oncol. 2018;15:422‐442. 29643471 10.1038/s41571-018-0003-5

[3] Di Mascolo D , Palange AL , Primavera R , et al. Conformable hierarchically engineered polymeric micromeshes enabling combinatorial therapies in brain tumours. Nat Nanotechnol. 2021;16(7):820‐829. 33795849 10.1038/s41565-021-00879-3

[4] Zou Y , Liu YJ , Yang ZP , Zhang DY , Lu YQ , Zheng M , Xue X , Geng J , Chung R , Shi BY . Effective and targeted human orthotopic glioblastoma xenograft therapy via a multifunctional biomimetic nanomedicine. Adv Mater. 2018;30(51):1803717. 10.1002/adma.201803717 30328157

[5] Chin L . Comprehensive genomic characterization defines human glioblastoma genes and core pathways (vol 455, pg 1061, 2008). Nature. 2013;494:506. 10.1038/nature07385 PMC2671642 18772890

[6] Wick W , Gorlia T , Bendszus M , et al. Lomustine and bevacizumab in progressive glioblastoma. New Engl J Med. 2017;377:1954‐1963. 29141164 10.1056/NEJMoa1707358

[7] Zhou M , Zhang ZY , Zhao HQ , Bao SQ , Cheng L , Sun J . An Immune‐related six‐lncRNA signature to improve prognosis prediction of glioblastoma multiforme. Mol Neurobiol. 2018;55:3684‐3697. 28527107 10.1007/s12035-017-0572-9

[8] Vander Heiden MG , Cantley LC , Thompson CB . Understanding the Warburg effect: the metabolic requirements of cell proliferation. Science. 2009;324:1029‐1033. 19460998 10.1126/science.1160809 PMC2849637

[9] Zhou K , Yao YL , He ZC , et al. VDAC2 interacts with PFKP to regulate glucose metabolism and phenotypic reprogramming of glioma stem cells. Cell Death Dis. 2018;9(10). 10.1038/s41419-018-1015-x PMC6155247 30250190

[10] Santos LC , Vogel R , Chipuk JE , Birtwistle MR , Stolovitzky G , Meyer P . Mitochondrial origins of fractional control in regulated cell death. Nat Commun. 2019;10(1):3687–3691. 30899020 10.1038/s41467-019-09275-x PMC6428895

[11] Viale A , Corti D , Draetta GF . Tumors and mitochondrial respiration: a neglected connection. Can Res. 2015;75:3687‐3691. 10.1158/0008-5472.CAN-15-0491 26374463

[12] Bonnay F , Veloso A , Steinmann V , et al. Oxidative metabolism drives immortalization of neural stem cells during tumorigenesis. Cell. 2020;182:1490‐1507.e19. 32916131 10.1016/j.cell.2020.07.039

[13] Iwata R , Casimir P , Vanderhaeghen P . Mitochondrial dynamics in postmitotic cells regulate neurogenesis. Science. 2020;369:858‐862. 32792401 10.1126/science.aba9760

[14] Le A , Stine ZE , Nguyen C , et al. Tumorigenicity of hypoxic respiring cancer cells revealed by a hypoxia‐cell cycle dual reporter. Proc Natl Acad Sci USA. 2014;111:12486‐12491. 25114222 10.1073/pnas.1402012111 PMC4151727

[15] Viale A , Corti D , Draetta GF . Tumors and mitochondrial respiration: a neglected connection. Can Res. 2015;75:3685‐3686. 10.1158/0008-5472.CAN-15-0491 26374463

[16] Missiroli S , Bonora M , Patergnani S , et al. PML at mitochondria‐associated membranes is critical for the repression of autophagy and cancer development. Cell Rep. 2016;16:2415‐2427. 27545895 10.1016/j.celrep.2016.07.082 PMC5011426

[17] Onorati AV , Dyczynski M , Ojha R , Amaravadi RK . Targeting autophagy in cancer. Cancer. 2018;124:3307‐3318. 29671878 10.1002/cncr.31335 PMC6108917

[18] Zheng Z , Zhang T , Liu H , et al. Bright near‐infrared aggregation‐induced emission luminogens with strong two‐photon absorption, excellent organelle specificity, and efficient photodynamic therapy potential. ACS Nano. 2018;12:8145‐8159. 30074773 10.1021/acsnano.8b03138

[19] Xie YQ , Li Q , Yang QB , et al. Overexpression of DCF1 inhibits glioma through destruction of mitochondria and activation of apoptosis pathway. Sci Rep. 2014;4:3702–3709. 24424470 10.1038/srep03702 PMC3892183

[20] Wang J , Wang Q , Zhou FF , et al. The antitumor effect of TAT‐DCF1 peptide in glioma cells. Neuropeptides. 2018;71:21‐31. 30001801 10.1016/j.npep.2018.06.004

[21] Liu Q , Chen Y , Li Q , Wu L , Wen T . Dcf1 regulates neuropeptide expression and maintains energy balance. Neurosci Lett. 2017;650:1‐7. 28377324 10.1016/j.neulet.2017.03.052

[22] Boada‐Romero E , Letek M , Fleischer A , Pallauf K , Ramon‐Barros C , Pimentel‐Muinos FX . TMEM59 defines a novel ATG16L1‐binding motif that promotes local activation of LC3. The EMBO Journal. 2013;32:566‐582. 23376921 10.1038/emboj.2013.8 PMC3579146

[23] Hamaoui D , Cosse MM , Mohan J , Lystad AH , Wollert T , Subtil A . The Chlamydia effector CT622/TaiP targets a nonautophagy related function of ATG16L1. Proc Natl Acad Sci USA. 2020;117:26784‐26794. 33055216 10.1073/pnas.2005389117 PMC7604492

[24] Boada‐Romero E , Serramito‐Gomez I , Sacristan MP , Boone DL , Xavier RJ , Pimentel‐Muinos FX . The T300A Crohn's disease risk polymorphism impairs function of the WD40 domain of ATG16L1. Nat Commun. 2016;7:11821. 27273576 10.1038/ncomms11821 PMC4899871

[25] Gerlach JP , Jordens I , Tauriello DVF , et al. TMEM59 potentiates Wnt signaling by promoting signalosome formation. Proc Natl Acad Sci USA. 2018;115:E3996‐E4005. 29632210 10.1073/pnas.1721321115 PMC5924918

[26] Wen LWJWYWJWSPRPTW . A novel function of dcf1 during the differentiation.pdf. Cell Mol Neurobiol. 2008:28. 10.1007/s10571-008-9266-1 PMC11515049 18365309

[27] Wang J , Li J , Wang Q , et al. Dcf1 deficiency attenuates the role of activated microglia during neuroinflammation. Front Mol Neurosci. 2018;11:256. 30104955 10.3389/fnmol.2018.00256 PMC6077288

[28] Chen YL , Feng RL , Luo GH , et al. DCF1 subcellular localization and its function in mitochondria. Biochimie. 2018;144:50‐55. 29074393 10.1016/j.biochi.2017.10.013

[29] Wang J , Wang F , Li Q , et al. Proteomics and molecular network analyses reveal that the interaction between the TAT‐DCF1 peptide and TAF6 induces an antitumor effect in glioma cells. Mol Omics. 2020;16:73‐82. 31899468 10.1039/c9mo00068b

[30] Rape M . Ubiquitylation at the crossroads of development and disease. Nat Rev Mol Cell Bio. 2018;19:59‐70. 28928488 10.1038/nrm.2017.83

[31] Schwertman P , Bekker‐Jensen S , Mailand N . Regulation of DNA double‐strand break repair by ubiquitin and ubiquitin‐like modifiers. Nat Rev Mol Cell Bio. 2016;17:379‐394. 27211488 10.1038/nrm.2016.58

[32] Changou CA , Chen YR , Xing L , et al. Arginine starvation‐associated atypical cellular death involves mitochondrial dysfunction, nuclear DNA leakage, and chromatin autophagy. Proc Natl Acad Sci USA. 2014;111:14147‐14152. 25122679 10.1073/pnas.1404171111 PMC4191793

[33] Kiriyama Y , Nochi H . Intra‐ and intercellular quality control mechanisms of mitochondria. Cells. 2017;7. 10.3390/cells7010001 PMC5789274 29278362

[34] Zong WX , Rabinowitz JD , White E . Mitochondria and cancer. Mol Cell. 2016;61:667‐676. 26942671 10.1016/j.molcel.2016.02.011 PMC4779192

[35] LeBleu VS , O'Connell JT , Gonzalez Herrera KN , et al. PGC‐1alpha mediates mitochondrial biogenesis and oxidative phosphorylation in cancer cells to promote metastasis. Nat Cell Biol. 2014;16(992–1003):1001‐1015. 10.1038/ncb3039 PMC4369153 25241037

[36] Ploumi C , Daskalaki I , Tavernarakis N . Mitochondrial biogenesis and clearance: a balancing act. FEBS J. 2017;284:183‐195. 27462821 10.1111/febs.13820

[37] Ghavami S , Shojaei S , Yeganeh B , et al. Autophagy and apoptosis dysfunction in neurodegenerative disorders. Prog Neurogibol. 2014;112:24‐49. 10.1016/j.pneurobio.2013.10.004 24211851

[38] Ma K , Wu H , Li P , Li B . LC3‐II may mediate ATR‐induced mitophagy in dopaminergic neurons through SQSTM1/p62 pathway. Acta Biochim Biophys Sin. 2018;50(10):1047‐1061. 30084861 10.1093/abbs/gmy091

[39] Marquez RT , Xu L . Bcl‐2: Beclin 1 complex: multiple, mechanisms regulating autophagy/apoptosis toggle switch. Am J Cancer Res. 2012;2:214‐221. 22485198 PMC3304572

[40] Rebecca T , Marquez LX . Bcl‐2‐Beclin 1 complex‐multiple, mechanisms regulating autophagy‐apoptosis toggle switch. Am J Cancer Res. 2012;2:214‐221. 22485198 PMC3304572

[41] Lazarou M , Sliter DA , Kane LA , et al. The ubiquitin kinase PINK1 recruits autophagy receptors to induce mitophagy. Nature. 2015;524:309‐314. 26266977 10.1038/nature14893 PMC5018156

[42] Nazio F , Strappazzon F , Antonioli M , et al. mTOR inhibits autophagy by controlling ULK1 ubiquitylation, self‐association and function through AMBRA1 and TRAF6. Nat Cell Biol. 2013;15:406‐416. 23524951 10.1038/ncb2708

[43] Yang Y , Zong Y , Sun Q , Jia Y , Zhao R . White light emitting diode suppresses proliferation and induces apoptosis in hippocampal neuron cells through mitochondrial cytochrome c oxydase‐mediated IGF‐1 and TNF‐alpha pathways. Free Radic Biol Med. 2017;113:413‐423. 29106990 10.1016/j.freeradbiomed.2017.10.382

[44] Levine B , Kroemer G . Autophagy in the pathogenesis of disease. Cell. 2008;132:27‐42. 18191218 10.1016/j.cell.2007.12.018 PMC2696814

[45] Mathew R , Karantza‐Wadsworth V , White E . Role of autophagy in cancer. Nat Rev Cancer. 2007;7:961‐967. 17972889 10.1038/nrc2254 PMC2866167

[46] Mellman I . Organelles observed: lysosomes. Science. 1989;244:853‐854. 17802262 10.1126/science.244.4906.853

[47] Sardiello M , Palmieri M , di Ronza A , et al. A gene network regulating lysosomal biogenesis and function. Science. 2009;325:473‐477. 19556463 10.1126/science.1174447

[48] Settembre C , Di Malta C , Polito VA , et al. TFEB links autophagy to lysosomal biogenesis. Science. 2011;332:1429‐1433. 21617040 10.1126/science.1204592 PMC3638014

[49] Yu LI , McPhee CK , Zheng L , et al. Termination of autophagy and reformation of lysosomes regulated by mTOR. Nature. 2010;465:942‐946. 20526321 10.1038/nature09076 PMC2920749

[50] Zhou J , Tan SH , Nicolas V , et al. Activation of lysosomal function in the course of autophagy via mTORC1 suppression and autophagosome‐lysosome fusion. Cell Res. 2013;23:508‐523. 23337583 10.1038/cr.2013.11 PMC3616426

[51] Bustos V , Pulina MV , Bispo A , et al. Phosphorylated Presenilin 1 decreases β‐amyloid by facilitating autophagosome‐lysosome fusion. Proc Natl Acad Sci USA. 2017:201705240. 10.1073/pnas.1705240114 PMC5502640 28533369

[52] Eskelinen E‐L , Schmidt CK , Neu S , et al. Disturbed cholesterol traffic but normal proteolytic function in LAMP‐1/LAMP‐2 double‐deficient fibroblasts. Mol Biol Cell. 2004;15:3132‐3145. 15121881 10.1091/mbc.E04-02-0103 PMC452571

[53] Huynh KK , Eskelinen EL , Scott CC , Malevanets A , Saftig P , Grinstein S . LAMP proteins are required for fusion of lysosomes with phagosomes. EMBO J. 2007;26:313‐324. 17245426 10.1038/sj.emboj.7601511 PMC1783450

[54] Hao M , Yeo SK , Turner K , et al. Autophagy blockade limits HER2+ breast cancer tumorigenesis by perturbing HER2 trafficking and promoting release via small extracellular vesicles. Dev Cell. 2021;56(341–355):e345. 10.1016/j.devcel.2020.12.016 33472043

[55] Kim BJ , Chan DW , Jung SY , Chen Y , Qin J , Wang Y . The histone variant MacroH2A1 Is a BRCA1 ubiquitin ligase substrate. Cell Rep. 2017;19:1758‐1766. 28564596 10.1016/j.celrep.2017.05.027 PMC6507409

[56] Draberova E , D'Agostino L , Caracciolo V , et al. Overexpression and nucleolar localization of gamma‐tubulin small complex proteins GCP2 and GCP3 in glioblastoma. J Neuropathol Exp Neurol. 2015;74:723‐742. 26079448 10.1097/NEN.0000000000000212

[57] Chen Q , Cai JQ , Wang QX , et al. Long noncoding RNA NEAT1, regulated by the EGFR pathway, contributes to glioblastoma progression through the WNT/beta‐catenin pathway by scaffolding EZH2. Clin Cancer Res. 2018;24:684‐695. 29138341 10.1158/1078-0432.CCR-17-0605

[58] Sanchez‐Vega F , Mina M , Armenia J , et al. Oncogenic signaling pathways in the cancer genome atlas. Cell. 2018;173:321‐337.e10. 29625050 10.1016/j.cell.2018.03.035 PMC6070353

[59] Zhang M , Huang N , Yang X , et al. A novel protein encoded by the circular form of the SHPRH gene suppresses glioma tumorigenesis. Oncogene. 2018;37:1805‐1814. 29343848 10.1038/s41388-017-0019-9

[60] Thorburn J , Horita H , Redzic J , Hansen K , Frankel AE , Thorburn A . Autophagy regulates selective HMGB1 release in tumor cells that are destined to die. Cell Death Differ. 2009;16:175‐183. 18846108 10.1038/cdd.2008.143 PMC2605182

[61] Daw CC , Ramachandran K , Enslow BT , et al. Lactate elicits ER‐Mitochondrial Mg(2+) dynamics to integrate cellular metabolism. Cell. 2020;183(474–489):e417. 10.1016/j.cell.2020.08.049 PMC7572828 33035451

[62] Panigrahi DP , Praharaj PP , Bhol CS , et al. The emerging, multifaceted role of mitophagy in cancer and cancer therapeutics. Semin Cancer Biol. 2020;66:45‐58. 31351198 10.1016/j.semcancer.2019.07.015

