# Supplementary material for: Dcf1 induces glioblastoma cells apoptosis by blocking autophagy
Source: Cancer Med. 2021 Nov 19;11(1):207–23. doi: 10.1002/cam4.4440 (PMC8704163; doi:10.1002/cam4.4440)
Supplement: Supplementary file 1 — Supplementary Material [file CAM4-11-207-s001.docx]

### *Dcf1* Induces Glioblastoma Cells Apoptosis by Blocking Autophagy

Guanghong Luo, Ruili Feng, Wengang Li, Yanlu Chen, Yangyang Sun, Junfeng Ma, Yanhong Duo,TieqiaoWen

This word file includes:

Supplementary Fig 1~8

Supplementary Table 1~4

**Supplemental Figures legends**

**Figure S1.** The expression of *Dcf1* in glioblastoma cells. Isolated glioblastoma cells were transfected with pcDNA3.1 or pcDNA3.1-Dcf1 for 48 h, and different assays were conducted according to the manufacturers’ instructions. (A) RT-PCR analysis of *Dcf1* expression in normal and tumor tissues (n=6). (B) Western blotting analysis of DCF1 expression in normal and tumor tissues (n=3). (C) Gene microarray analysis of *Dcf1* expression in tumor tissues and cell lines. LG: low-grade glioblastoma (n=6), HG: high-grade glioblastoma (n=4), NA: normal astrocyte (n=3). (D) Alignment of the *Dcf1* sequence in tumor tissue with that in the NCBI database (ID: NM 004872.4). *Dcf1* showed no mutations. Data were presented as mean ± SEM. Significance between every two groups was calculated by the Student’s t-test. *P < 0.05, **P < 0.01, ***P < 0.001.

**Figure S2.** Identification of the iTRAQ sequence and bioinformatic analysis of DEPs. (A) Downregulated proteins upon *Dcf1* overexpression. (B) Upregulated proteins upon *Dcf1* overexpression. (C) Kyoto Encyclopedia of Genes and Genomes (KEGG) analysis of the DEPs (top 20). (D) Search Tool for the Retrieval of Interacting Genes/Proteins (STRING) analysis of HistoneH2A. (E) Western blotting detection of UBA52 (n=4). Data were presented as mean ± SEM. Significance between every two groups was calculated by the Student’s t-test. *P < 0.05, **P < 0.01, ***P < 0.001.

**Figure S3.** Summary of the localization of DEPs. (A) Summary of differentiated proteins in major organelles. (B) Summary of up- and downregulated proteins in different organelles. (C) Immunofluorescence image of intracellular Ca^2+^.

**Figure S4.** *Dcf1* promoted mitophagy. (A) Colocalization immunofluorescence image of Bcl-2 and BECN1 at different time points. (B) Ratio between BECN1 and Bcl-2 determined by Western blotting at different time intervals (n=3). (C) Western blotting of mitophagy markers (n=3). (D) Western blotting results for the mitophagy pathway (n=3). Scale bars: 50 μm. Data were presented as mean ± SEM. Significance between every two groups was calculated by the Student’s t-test. *P < 0.05, **P < 0.01, ***P < 0.001.

**Figure S5.** *Dcf1* affected the function of lysosomes. (A) Examination of endosome markers in glioblastoma tumor tissue by Western blotting (n=4). (B) Endosome levels determined by Western blotting after *Dcf1* overexpression (n=4). (C) Immunofluorescence image of LAMP1. (D) Immunofluorescence image of Cathepsin B in cytosol. (E) Immunofluorescence image of Cathepsin D in the cytosol. Scale bars: 50 μm. Data were presented as mean ± SEM. Significance between every two groups was calculated by the Student’s t-test. *P < 0.05, **P < 0.01, ***P < 0.001.

**Figure S6. *Dcf1* inhibited the viability of glioblastoma cells**. Isolated glioblastoma cells were transfected with pcDNA3.1 or pcDNA3.1-Dcf1 for 48 h, and different assays were conducted according to the manufacturers’ instructions. (**A**) The cell proliferation rate of glioblastoma cells was significantly inhibited by *Dcf1* overexpression, as determined by CCK-8 assay (n=6). (**B**) *Dcf1* inhibited the migration of glioblastoma cells (n=4). (**C**) *Dcf1* promoted the adhesion of glioblastoma cells with type I collagen (n=4). (**D**) *Dcf1* decreased the percentage of invasive glioblastoma cells (n=4). (E) Detection of the migration rate of GBM cells using xCELLigence RTCA DPlus (n=3). Red: glioblastoma cells transfected with pcDNA3.1, green: glioblastoma cells transfected with pcDNA3.1-Dcf1. (F) Detection of the cell cycle with a flow cytometer. (G) *Dcf1* significantly induced glioblastoma apoptosis (n=6). (**H**) Apoptotic detection of glioblastoma cells with a One Step TUNEL Apoptosis Assay Kit (n=4). Scale bar: 50 μm. Data were presented as mean ± SEM. Data were presented as mean ± SEM. Significance between every two groups was calculated by the Student’s t-test. *P < 0.05, **P < 0.01, ***P < 0.001.

**Figure S7.** *Dcf1* failed to induce the glioblastoma cells into senescence. (A) Brightfield image of glioblastoma cells transfected with pcDNA3.1 or pcDNA3.1-Dcf1. (B) Western blotting detection of cancer cellular senescence markers (n=4). (C) Western blotting detection of apoptotic proteins (n=4). Data were presented as mean ± SEM. Significance between every two groups was calculated by the Student’s t-test. *P < 0.05, **P < 0.01, ***P < 0.001.

**Figure S8.** Western blotting detection of apoptotic proteins via the extrinsic death receptor apoptosis pathway (n=4). Data were presented as mean ± SEM. Significance between every two groups was calculated by the Student’s t-test. *P < 0.05, **P < 0.01, ***P < 0.001.

**Supplemental Figures**


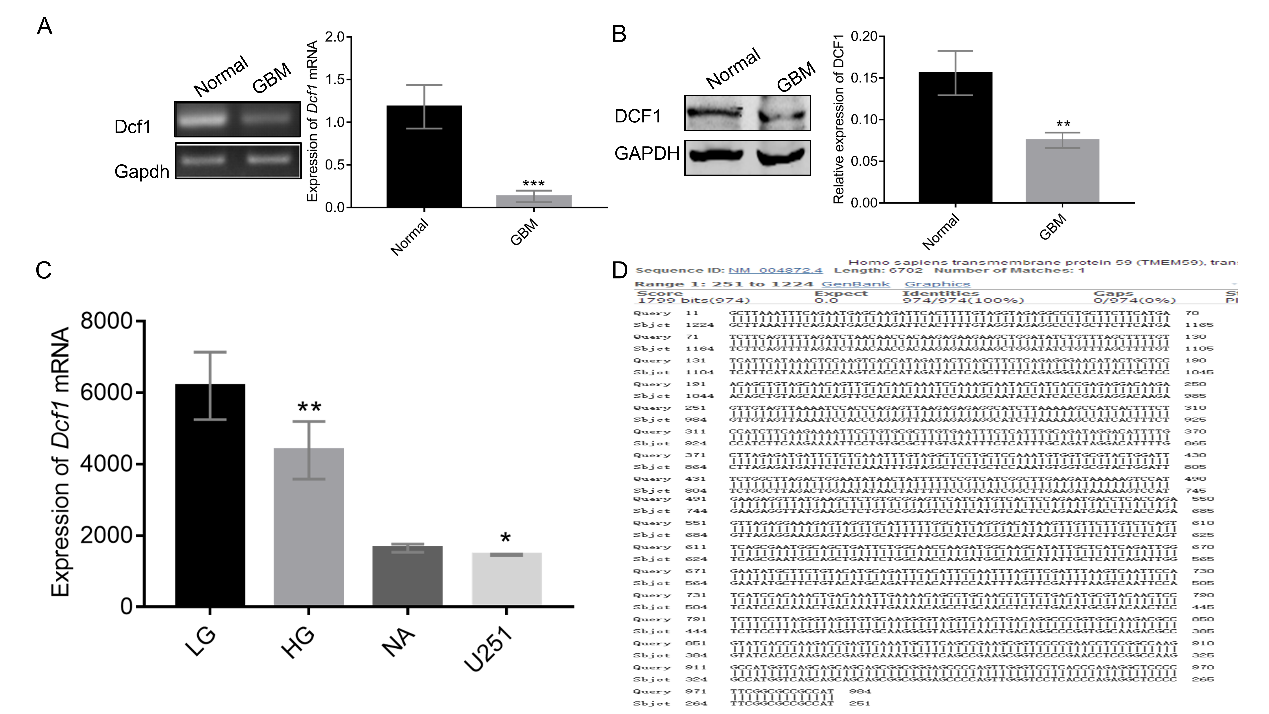


**Figure S2**


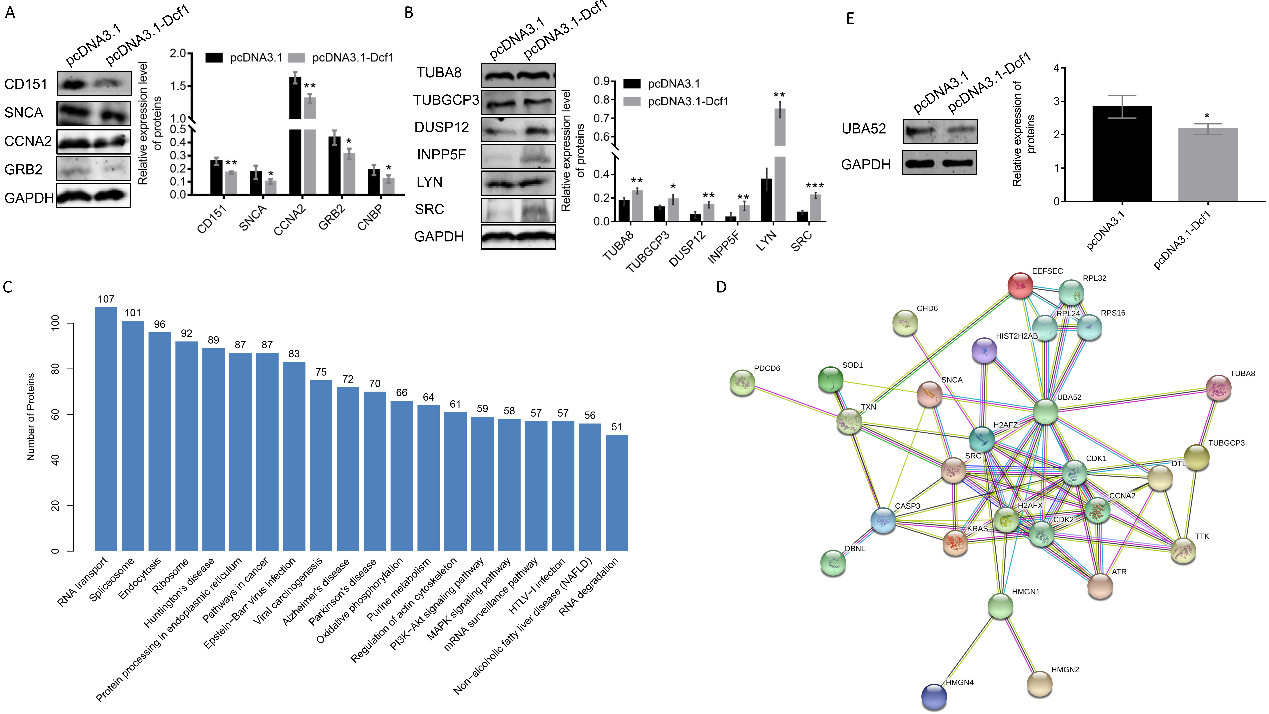


**Figure S3**


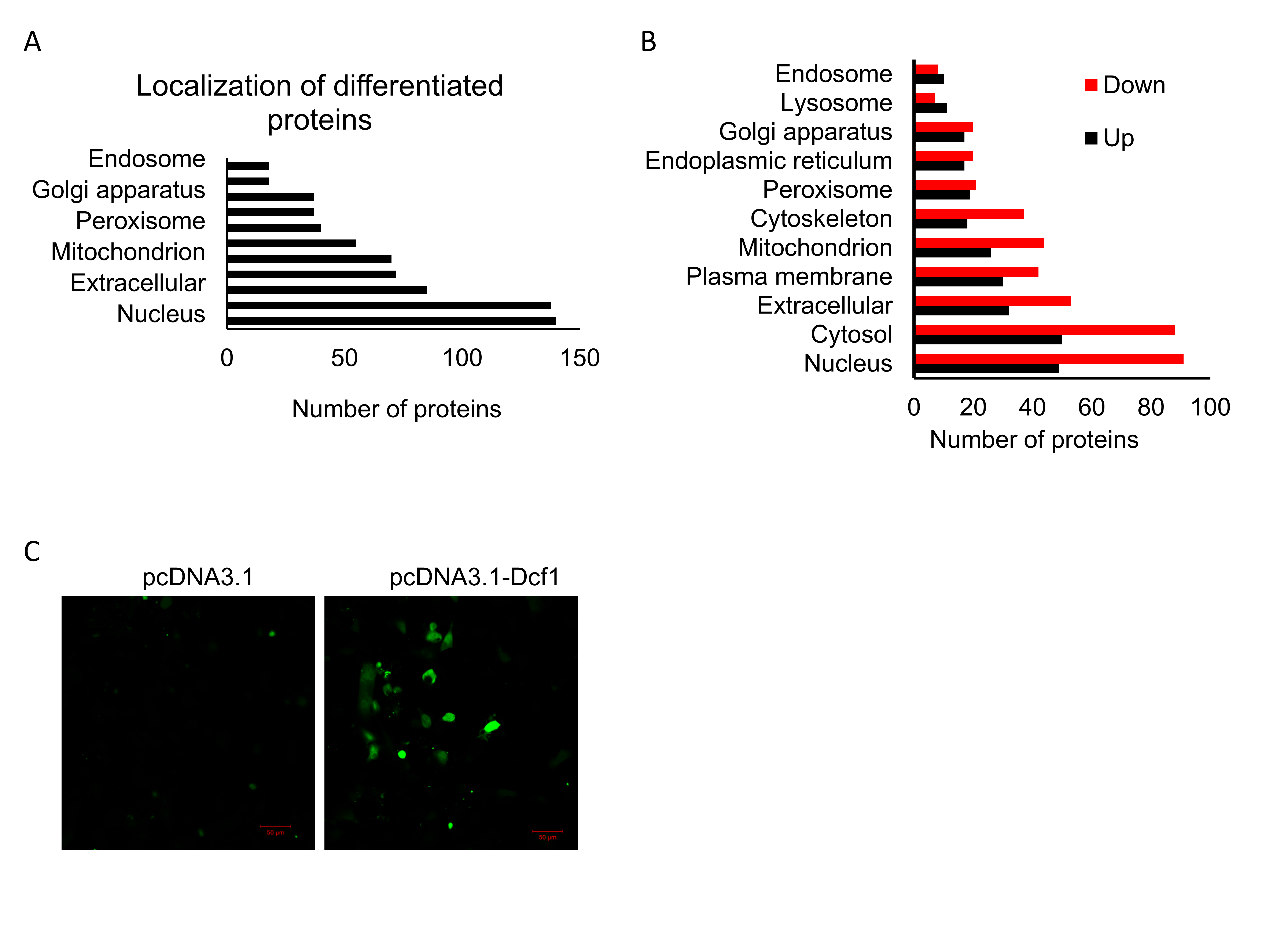


**Figure S4**


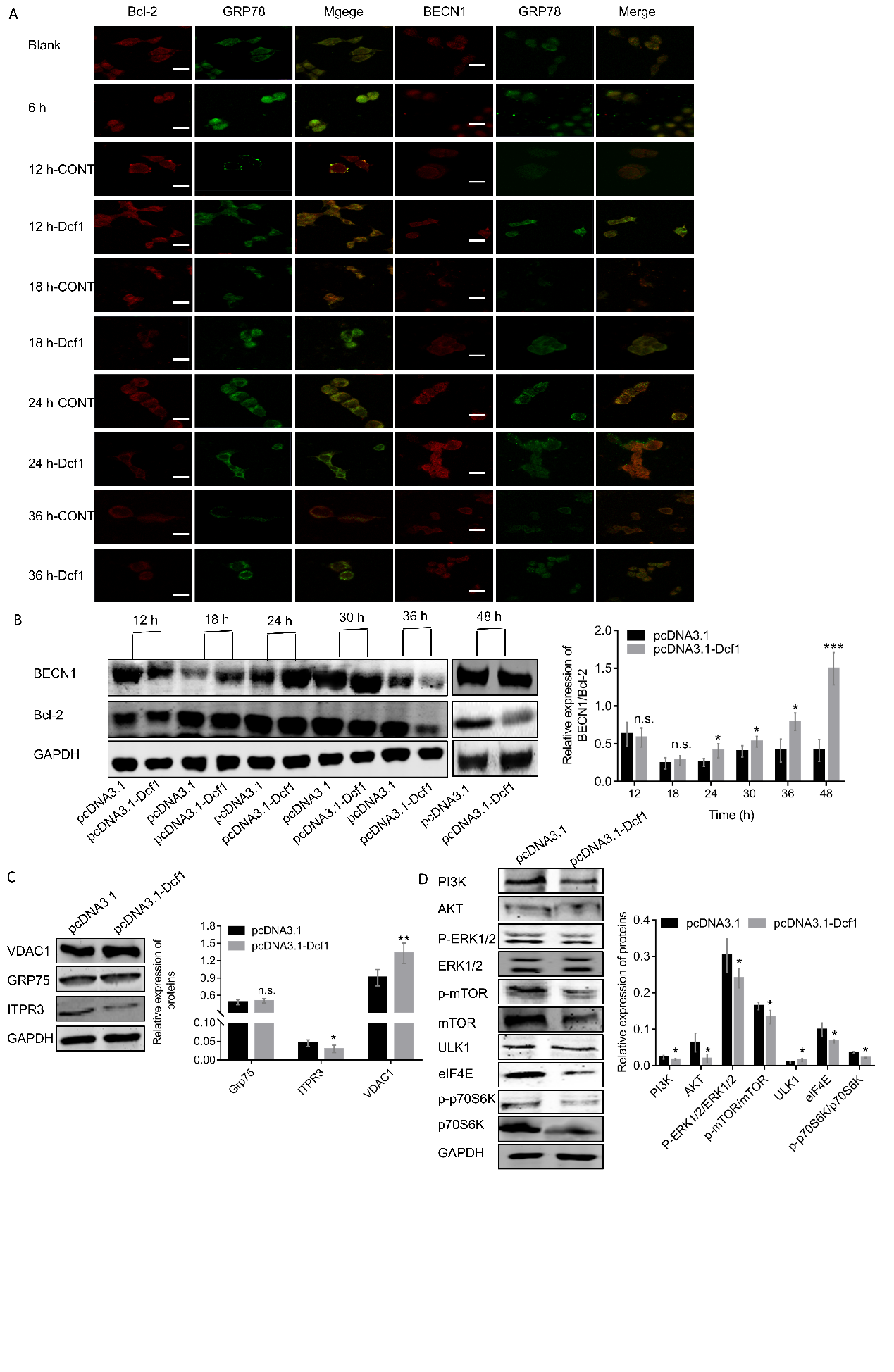


**Figure S5**


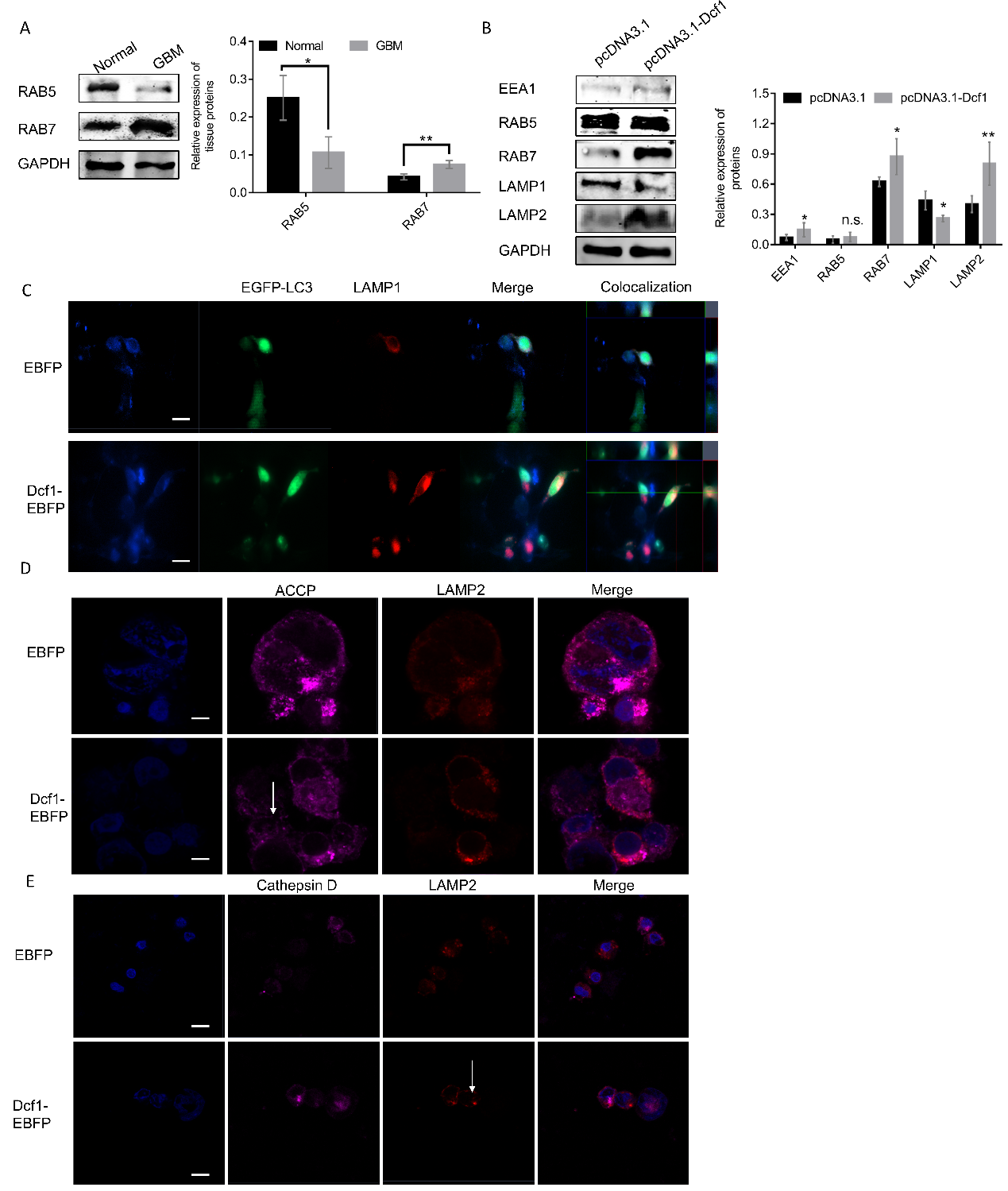


**Figure S6**


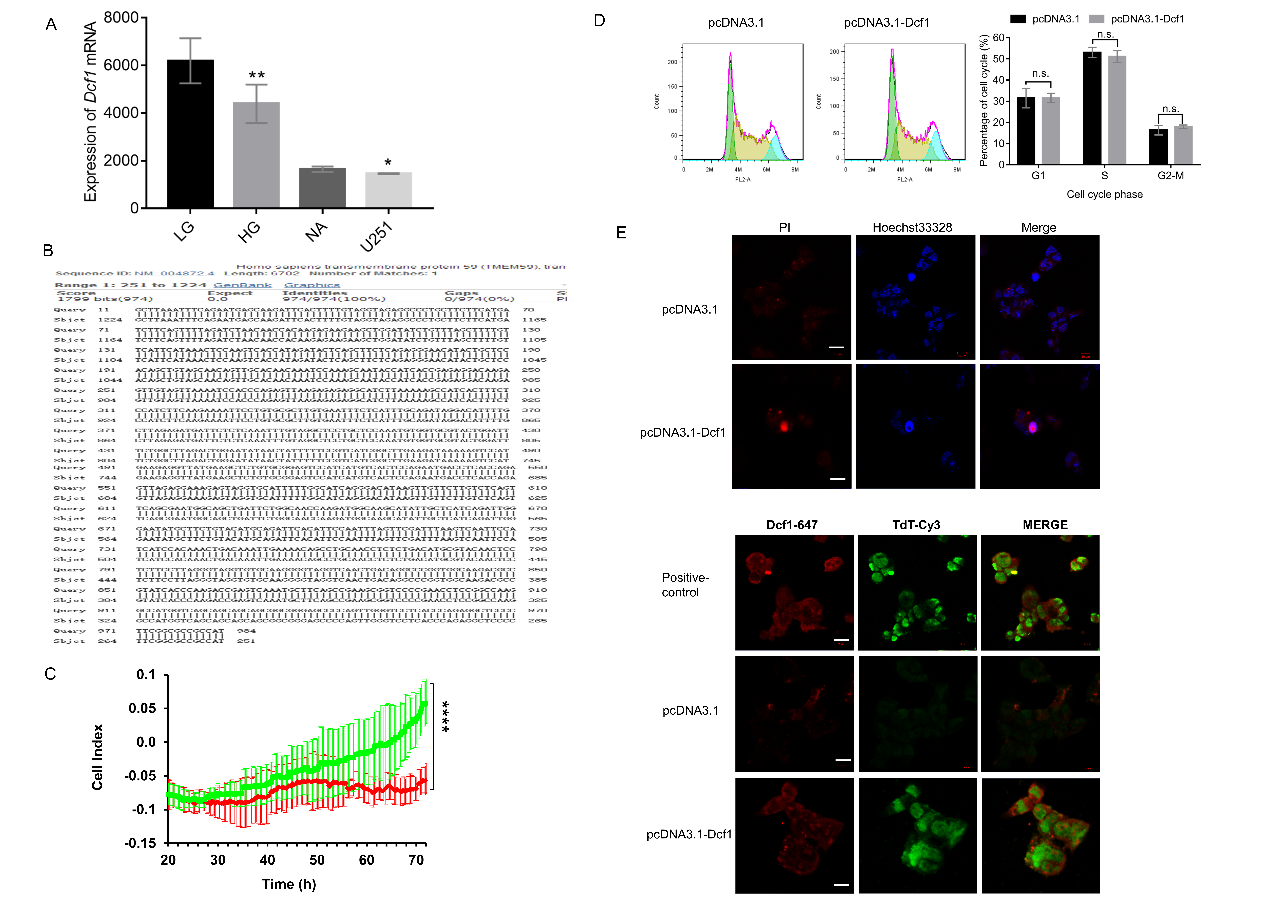


**Figure S7**


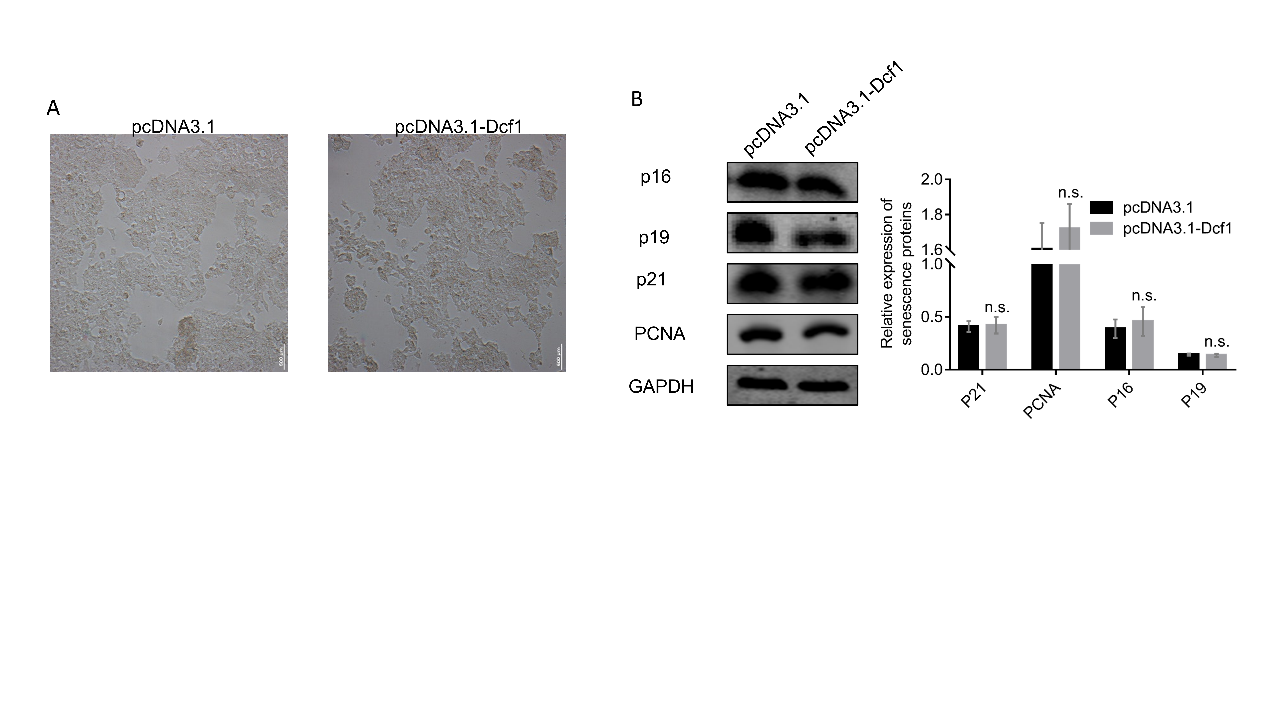


**Figure S8**


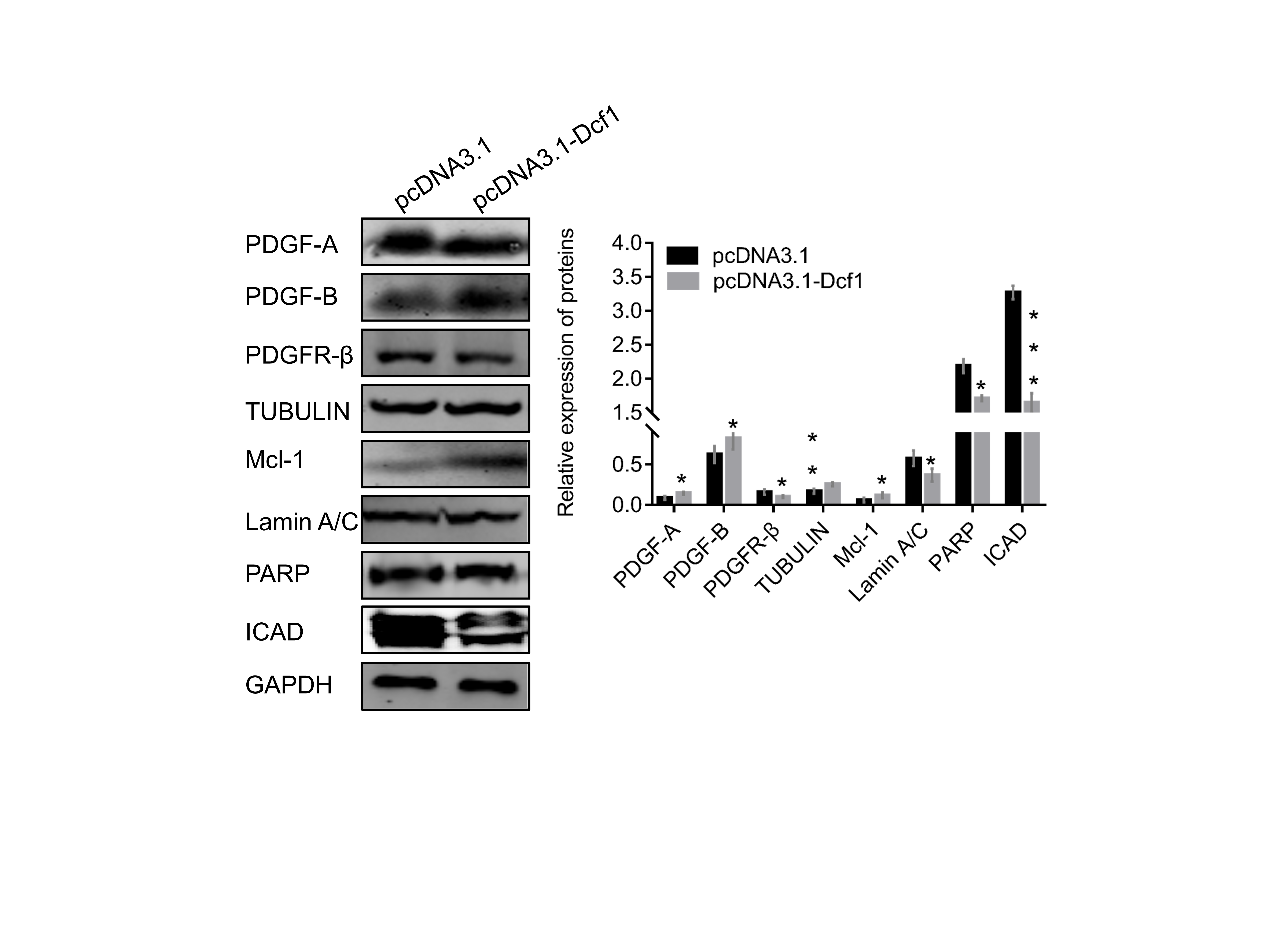


**Supplemental Tables**

**Supplemental Table 1.** The antibodies’ information.

| REAGENT or RESOURCE | IDENTIDFIER | HOST | SOURCE |
| --- | --- | --- | --- |
| Antibodies |  |  |  |
| p-mTOR | SC-293133 | M | SANTA CRUZ |
| HistoneH2A.X | SC-517336 | M | SANTA CRUZ |
| Akt1 | SC-5298 | M | SANTA CRUZ |
| survivin | SC-17779 | M | SANTA CRUZ |
| BID | SC-373939 | M | SANTA CRUZ |
| MTOR | SC-293089 | M | SANTA CRUZ |
| V-ATPase | SC-374475 | M | SANTA CRUZ |
| Fas | SC-8009 | M | SANTA CRUZ |
| SQSTM1 | SC-28359 | M | SANTA CRUZ |
| TP53 | SC-126 | M | SANTA CRUZ |
| PARP-1 | SC-8007 | M | SANTA CRUZ |
| GAPDH | SC-32233 | M | SANTA CRUZ |
| Optineurin | SC-166576 | M | SANTA CRUZ |
| GCP3 | SC-373758 | M | SANTA CRUZ |
| PDGF-α | SC-9974 | M | SANTA CRUZ |
| TRADD | SC-46653 | M | SANTA CRUZ |
| PDGF-β | SC-374573 | M | SANTA CRUZ |
| Granzyme b | SC-8022 | M | SANTA CRUZ |
| PI3K | SC-293172 | M | SANTA CRUZ |
| Calcoco2 | SC-376540 | M | SANTA CRUZ |
| p-TP53 | SC-377553 | M | SANTA CRUZ |
| FADD | SC-271748 | M | SANTA CRUZ |
| LKB1 | SC-32245 | M | SANTA CRUZ |
| PDGF-B | SC-365805 | M | SANTA CRUZ |
| ULK1 | SC-390904 | M | SANTA CRUZ |
| TRAIL | SC-78440 | M | SANTA CRUZ |
| ICAD | SC-17816 | M | SANTA CRUZ |
| PTEN | SC-7974 | M | SANTA CRUZ |
| AKT1 | SC-5298 | M | SANTA CRUZ |
| p53 | SC-126 | M | SANTA CRUZ |
| GRB2 | SC-8034 | M | SANTA CRUZ |
| INPP5F | SC-514657 | M | SANTA CRUZ |
| LYN | SC-7274 | M | SANTA CRUZ |
| LC3 | SC-398822 | M | SANTA CRUZ |
| PDGFRA | SC-398206 | M | SANTA CRUZ |
| SRC-1 | SC-32789 | M | SANTA CRUZ |
| P70S6K | SC-8418 | M | SANTA CRUZ |
| DUSP12 | SC-390760 | M | SANTA CRUZ |
| eIF4E | SC-271480 | M | SANTA CRUZ |
| DR4 | SC-8411 | M | SANTA CRUZ |
| CD151 | SC-271216 | M | SANTA CRUZ |
| GAP-43 | SC-17790 | M | SANTA CRUZ |
| NBR1 | SC-130380 | M | SANTA CRUZ |
| BECN1 | SC-48341 | M | SANTA CRUZ |
| AIF | SC-13116 | M | SANTA CRUZ |
| Presebilin1 | SC-365450 | M | SANTA CRUZ |
| P-P70S6K | SC-377529 | M | SANTA CRUZ |
| RAB5 | SC-46692 | M | SANTA CRUZ |
| LAMIN A/C | SC-376248 | M | SANTA CRUZ |
| RAB7 | SC-376362 | M | SANTA CRUZ |
| LAMP-2 | SC-18822 | M | SANTA CRUZ |
| Cathepsin B | SC-365558 | M | SANTA CRUZ |
| ACTIN | SC-8432 | M | SANTA CRUZ |
| LAMP-1 | SC-20011 | M | SANTA CRUZ |
| VDAC1 | SC-390996 | M | SANTA CRUZ |
| PPARGC1A | A12348 | R | ABCLONAL |
| ACTB | AC026 | R | ABCLONAL |
| TFAM | A1926 | R | ABCLONAL |
| NFE2L2 | A11159 | R | ABCLONAL |
| GAPDH | AB0037 | R | ABWAYS |
| Caspase-8 | A0215 | R | ABCLONAL |
| LC3 | A11282 | R | ABCLONAL |
| p-HistoneH2A.X-S139 | AP0099 | R | ABCLONAL |
| p-ERK1/2 | 8544S | R | CST |
| ERK1/2 | 4695S | M | CST |
| PPARGC1B | A17257 | R | ABCLONAL |
| SIRT3 | A7307 | R | ABCLONAL |
| SIRT1 | A11267 | R | ABCLONAL |
| NFE2L1 | A14753 | R | ABCLONAL |
| Bcl-2 | A2845 | R | ABCLONAL |
| IDH1 | 12332-1-AP | R | PROTEINTECH |
| Caspase-3 | A0214 | R | ABCLONAL |
| AIF | CY5024 | R | ABWAYS |
| RAF1 | A0223 | R | ABCLONAL |
| Survivin | D221289 | R | SANGON |
| Caspase-8 | A0215 | R | ABCLONAL |
| PARL | A8231 | R | ABCLONAL |
| ACPP | 15840-1-AP | R | PROTEINTECH |
| Cathepsin D | 21327-1-AP | R | PROTEINTECH |
| LAMP1 | A2582 | R | ABCLONAL |
| ITPR3 | D161805 | R | SANGON |
| TFEB | 13372-1-AP | R | PROTEINTECH |
| LC3 | CY5992 | R | ABWAYS |
| DCF1 |  |  | SELF-PROVIDE |
| Dylight 800 goat anti-rabbit immunoglobulin (Ig)G | 5230-0412 | N/A | KPL |
| DyLight 700 goat anti-mouse immunoglobulin (Ig)G | 072-06-18-06 | N/A | KPL |
| Alexa Fluor® 594-Cconjugated goat anti-mouse IgG (H+L) | ZF-0511 | N/A | Beijing Zhongshan Golden Bridge Bio-technology |
| Alexa Fluor® 488-Cconjugated goat anti-mouse IgG (H+L) | ZF-0512 | N/A | Beijing Zhongshan Golden Bridge Bio-technology |
| Alexa Fluor® 594-Cconjugated goat anti-mouse IgG (H+L) | ZF-0513 | N/A | Beijing Zhongshan Golden Bridge Bio-technology |
| Alexa Fluor® 488-Cconjugated goat anti-rabbit IgG (H+L) | ZF-0516 | N/A | Beijing Zhongshan Golden Bridge Bio-technology |
| Alexa Fluor® 647-Cconjugated donkey anti-mouse IgG (H+L) | 706-605-150 | N/A | KPL |

**Supplemental Table 2.** The plasmids’ information.

The plasmid used in this study is preserved or constructed at our lab.

| plasmid | [antibiotic resistance](http://www.baidu.com/link?url=2Pjhx1m1jpFbF2lIX5_rJsJvqTWiSe21EdYwgvj_qBSoZlUloA8Wq1OTyeyFUzpfvUmU-MfN8w3ZhhLWc6mu6tIaxUtfiX7pnlKw4wuaXASZR8bLWF49APhtpSWyVLIe) | SOURCE |
| --- | --- | --- |
| pEGFP-LC3 | Ampicillin | N/A |
| pN2-COX8-DsRed | Kanamycin | N/A |
| pN2-Dcf1-EGFP | Kanamycin | N/A |
| pN1-Dcf1-DsRed | Kanamycin | N/A |
| pN1-DsRed | Kanamycin | N/A |
| pCDNA3.1 | Ampicillin | N/A |
| pN2-EGFP | Kanamycin | N/A |
| pN1-EBFP | Kanamycin | N/A |
| pN2-Grp75-EGFP | Kanamycin | N/A |
| pC1-DsRed | Kanamycin | N/A |
| pN2-COX8-EGFP | Kanamycin | N/A |
| pDLV-LAMP1-mCherry | Kanamycin | N/A |
| pN1-Dcf1-EBFP | Kanamycin | N/A |
| pmCherry-EGFP-LC3 | Kanamycin | N/A |
| pN1-Dcf1-EBFP | Kanamycin | N/A |

**Supplemental Table 3.** The primes’ information of RT-PCT.

| PRIME | SEQUENCE | SOURCE |
| --- | --- | --- |
| qhDcf1-up | 5’-GAAGAGGAGTTGTACGCATGTC3’ | Shanghai RuiDi Biological Technology Co.,Ltd. |
| qhDcf1-rev | 5’-GCAACCAAGATGGCAAGCAT-3’ | Shanghai RuiDi Biological Technology Co.,Ltd. |
| qGAPDH-up | 5’-GGAGCGAGATCCCTCCAAAAT-3’ | Shanghai RuiDi Biological Technology Co.,Ltd. |
| qGAPDH-rev | 5’-GGCTGTTGTCATACTTCTCATGG-3’ | Shanghai RuiDi Biological Technology Co.,Ltd. |
| qL394 | 5’-CACCAGCCTAACCAGATTTC-3’ | Shanghai RuiDi Biological Technology Co.,Ltd. |
| qH475 | 5’-GGGTTGTATTGATGAGATTAGT-3’ | Shanghai RuiDi Biological Technology Co.,Ltd. |
| qHBG1F | 5’-GCTTCTGACACAACTGTGTTCACTAGC-3’ | Shanghai RuiDi Biological Technology Co.,Ltd. |
| qHBG1R | 5’-CACCAACTTCATCCACGTTCACC-3’ | Shanghai RuiDi Biological Technology Co.,Ltd. |
| Dcf1-up | 5-’CAAGAATTCGCCACCATGGCGGCGCCGA  AGGGGAGCCTC-3’ | Shanghai RuiDi Biological Technology Co.,Ltd. |
| Dcf1-rev | 5’-caaGGATCCATGATGATGATGATGATGAATTT  CAGAATGAGCAAGATTCAC-3’ | Shanghai RuiDi Biological Technology Co.,Ltd. |

**Supplemental Table 4.** The Chemicals, Kits’ information.

| Chemicals, Kits | IDENTIDFIER | SOURCE |
| --- | --- | --- |
| Mito-Tracker Green | C1048 | Beyotime Biotechnology |
| JC-1 | C2006 | Beyotime Biotechnology |
| Cell Mitochondria Isolation Kit | C3601 | Beyotime Biotechnology |
| Cell Counting Kit-8 | C0038 | Beyotime Biotechnology |
| One Step TUNEL Apoptosis Assay Kit | C1086 | Beyotime Biotechnology |
| Lyso-Tracker Red | C1046 | Beyotime Biotechnology |
| Acid Phosphatase Assay Kit | P0326 | Beyotime Biotechnology |
| ATP Assay Kit | S0026 | Beyotime Biotechnology |
| Fluo-4 AM | S1060 | Beyotime Biotechnology |
| BCECF AM | S1006 | Beyotime Biotechnology |
| AO Staining Kit | E607307 | Sangon |
| Annexin V-FITC/PI Apoptosis Detection Kit | 40302ES20 | Shanghai Yi Sheng Biotechnology Co., Ltd. |
| BioCoat™ Matrigel™ Invasion Chamber | BD354480 | BD |
